# Kaposi’s sarcoma-associated herpesvirus vFLIP promotes MEndT to generate hybrid M/E state for tumorigenesis

**DOI:** 10.1371/journal.ppat.1009600

**Published:** 2021-12-22

**Authors:** Weikang Chen, Yao Ding, Dawei Liu, Zhengzhou Lu, Yan Wang, Yan Yuan

**Affiliations:** 1 Institute of Human Virology, Zhongshan School of Medicine, Sun Yat-Sen University, Guangzhou, China; 2 Department of Pathology, The First Affiliated Hospital, Sun Yat-sen University, Guangzhou, China; 3 Guanghua School of Stomatology, Guangdong Provincial Key Laboratory of Stomatology, Sun Yat-Sen University, Guangzhou, China; 4 Department of Basic and Translational Sciences, University of Pennsylvania School of Dental Medicine, Philadelphia, Pennsylvania, United States of America; Florida State University, UNITED STATES

## Abstract

Kaposi’s sarcoma (KS) is an angioproliferative and invasive tumor caused by Kaposi’s sarcoma-associated herpesvirus (KSHV). The cellular origin of KS tumor cells remains contentious. Recently, evidence has accrued indicating that KS may arise from KSHV-infected mesenchymal stem cells (MSCs) through mesenchymal-to-endothelial transition (MEndT), but the transformation process has been largely unknown. In this study, we investigated the KSHV-mediated MEndT process and found that KSHV infection rendered MSCs incomplete endothelial lineage differentiation and formed hybrid mesenchymal/endothelial (M/E) state cells characterized by simultaneous expression of mesenchymal markers Nestin/PDGFRA/α-SAM and endothelial markers CD31/PDPN/VEGFR2. The hybrid M/E cells have acquired tumorigenic phenotypes *in vitro* and the potential to form KS-like lesions after being transplanted in mice under renal capsules. These results suggest a homology of KSHV-infected MSCs with Kaposi’s sarcoma where proliferating KS spindle-shaped cells and the cells that line KS-specific aberrant vessels were also found to exhibit the hybrid M/E state. Furthermore, the genetic analysis identified KSHV-encoded FLICE inhibitory protein (vFLIP) as a crucial regulator controlling KSHV-induced MEndT and generating hybrid M/E state cells for tumorigenesis. Overall, KSHV-mediated MEndT that transforms MSCs to tumorigenic hybrid M/E state cells driven by vFLIP is an essential event in Kaposi’s sarcomagenesis.

## Author summary

Kaposi’s sarcoma manifests as multifocal lesions with spindle cell proliferation, intense angiogenesis, and erythrocyte extravasation. Although the origin and malignant nature of KS remain contentious, it is established that KSHV infection with concomitant viral oncogene expression in normal cell progenitors causes KS. The mechanism of KSHV oncogenesis could be revealed through a reproduction of KS by infection of normal cells. This study reports that the KSHV infection of mesenchymal stem cells initiates mesenchymal-to-endothelial transition (MEndT) that generates mesenchymal/endothelial (M/E) hybrid state cells. The hybrid M/E cells acquired tumorigenic phenotypes, including tumor initiation, angiogenesis, migration, and the potential to form KS-like lesions after transplanted in mice. This finding faithfully recapitulates Kaposi’s sarcoma where proliferating KS spindle cells and the cells that line KS-specific aberrant vessels are also found to exhibit the hybrid M/E phenotype. We also found that KSHV-encoded viral FLICE inhibitory protein (vFLIP) plays a crucial role in promoting MEndT and the generation of M/E state cells. These results provide a new layer of evidence for KSHV-infected MSCs being the cell source of KS spindle cells and reveal novel insight into KS pathogenesis and viral tumorigenesis.

## Introduction

Kaposi’s sarcoma (KS) is the most common neoplasm in AIDS patients. Kaposi’s sarcoma-associated herpesvirus (KSHV) is the causative agent of this malignancy [1]. KSHV is also associated with other malignancies, including primary effusion lymphoma (PEL) [2], multicentric Castleman’s disease (MCD) [3]. Recent reports also suggest an involvement of KSHV in childhood osteosarcoma [4]. Kaposi’s sarcoma is a multicentric, oligoclonal neoplasm clinically presenting as red-purplish spots localize mainly in the oral cavity or skin [5]. The histological features of KS lesions are extremely complex. They consist of proliferating spindle tumor cells, immature and leaky vessels, and prominent inflammatory infiltrate. The cellular origin of KS spindle cells remains controversial. The current leading hypothesis is that KS spindle cells may derive from endothelial lineage, as they bear pan-endothelial markers (CD31, CD34, and CD36 and Factor VIII) and lymphatic endothelial markers (VEGFR3, LYVE-1 and PDPN). However, KS spindle cells also express other markers including smooth muscle cell (α-SAM), macrophage (CD68), dendritic cell (Factor XIII) and mesenchymal stem cell (Nestin and CD29) markers, suggesting that KS cells do not faithfully represent an endothelial cell lineage [6]. Besides, KS spindle cells display intriguing characteristics of progenitor or immature endothelial cells—the expression of endothelial progenitor cell markers and lack of Weibel-Palade bodies (WPB) regarded as a marker for mature vascular endothelium [7,8]. The remarkable heterogeneity of KS raised a hypothesis that KS spindle cells may originate from mesenchymal stem cells (MSCs) or precursors of vascular cells [9,10]. Recently, we found a series of evidence supporting the hypothesis. (i) An immuno-histochemistry analysis showed that AIDS-KS spindle cells express Neuroectodermal stem cell marker (Nestin) and oral MSC marker CD29, suggesting an oral/craniofacial MSC lineage of AIDS-associated KS. (ii) KSHV infection of oral MSCs effectively promotes multiple lineage differentiation, especially endothelial differentiation *in vitro* and *in vivo*. (iii) Gene expression profiling analysis showed that KSHV infection reprograms MSCs, resulting in mesenchymal-to-endothelial transition (MEndT) and rendering KSHV-infected MSC the closest distance to Kaposi’s sarcoma in gene expression profile. (iv) When implanted in mice, KSHV-infected MSCs were transformed into KS-like spindle-shaped cells with other KS-like phenotypes [11]. Moreover, KSHV-infected primary rat embryonic metanephric mesenchymal precursor cells (KMM) and mouse bone marrow-derived MSCs (KPα(+)S) growth in KS-like conditions efficiently form KS-like tumors in nude mice [10,12]. Taken together, increasing evidence supports the notion that Kaposi’s sarcoma may arise from KSHV-infected MSCs through MEndT. However, the underlying mechanism remains unclear.

MSCs have been identified as a population of hierarchical postnatal stem cells with the potential to self-renew and differentiate into osteoblasts, chondrocytes, adipocytes, cardiomyocytes, myoblasts, and neural cells [13,14]. MSCs can be induced to endothelial-like cells with angiogenic cytokines, including VEGF, bFGF, and angiopoietin [15]. The switch from mesenchymal to endothelial phenotype is referred to as Mesenchymal-to-Endothelial Transition (MEndT), which is a critical phase of embryonic organic development and also contributes to diseases. In an adult, MEndT contributes to neovascularization by inducing cardiac fibroblasts to generate endothelial cells after cardiac injury [16], and to cancer progression by enhancing angiogenesis [17]. Moreover, MEndT, like its reverse process EndMT, is not a binary switch but a dynamic transition, which generates many intermediate phenotypic states arrayed along the mesenchymal (M)-to-endothelial (E) spectrum including mesenchymal-like (M), endothelial-like (E), and hybrid M/E states. Studies suggested that tumor cells staying in different stages have different roles in tumor progression [18–21].

KSHV can be found in all KS tumors and is present in all stages of KS (patch, plaque, and nodular). In the early (patch) stage, KSHV is found in spindle-like cells surrounding ectatic vessels, and in nodular KS, the virus is present in the vast majority of spindle cells surrounding slit-like vessels [22]. The majority of KSHV in KS lesions is in a latent phase where only a limited number of latent genes are expressed. In a small percentage of tumor cells, KSHV undergoes spontaneous lytic replication and expresses viral lytic genes. KSHV latency genes, including LANA, vCyclin, and vFLIP, are known to play roles in regulating cell proliferation and apoptosis evasion and endowing pro-angiogenic and inflammatory signals [6,23]. Some lytic viral proteins, such as vGPCR and vIL-6, exhibit tumorigenic activities and induce angiogenesis and inflammation [23–25]. vIL6 sufficiently induces BECs to differentiate to LECs via upregulating the expression of PROX1 [26], KSHV-encoded miRNAs induce LEC-to-BEC reprogramming via downregulating MAF [27], KSHV-initiated endothelial-to-mesenchymal transformation is mediated by vFLIP and vGPCR through MT1-MMP in 3D LECs culture system [28]. Thus, many viral genes are known to participate in KSHV-induced cell reprogramming and KS oncogenesis.

Increasing evidence supports that KS derives from KSHV-infected MSC through MEndT. However, how KSHV infection drives mesenchymal stem cells for the MEndT process that leads to Kaposi’s sarcoma was largely unknown. In this study, we investigated the process of KSHV-mediated MEndT and the underlying mechanism. We found that KSHV infection initiates an endothelial lineage, but incomplete differentiation that generates premalignant cells with hybrid mesenchymal and endothelial phenotypes. Such hybrid M/E cells exhibit oncogenic properties and form KS-like lesions in kidney capsule transplantation. Finally, KSHV vFLIP was found to play critical roles in KSHV-induced MEndT and oncogenesis. These findings further support the hypothesis that KS tumor cells arise from KSHV-infected MSCs through MEndT.

## Results

### KSHV-positive spindle cells in Kaposi’s sarcoma lesions display a hybrid mesenchymal/ endothelial (M/E) phenotype

**Fig 1:**
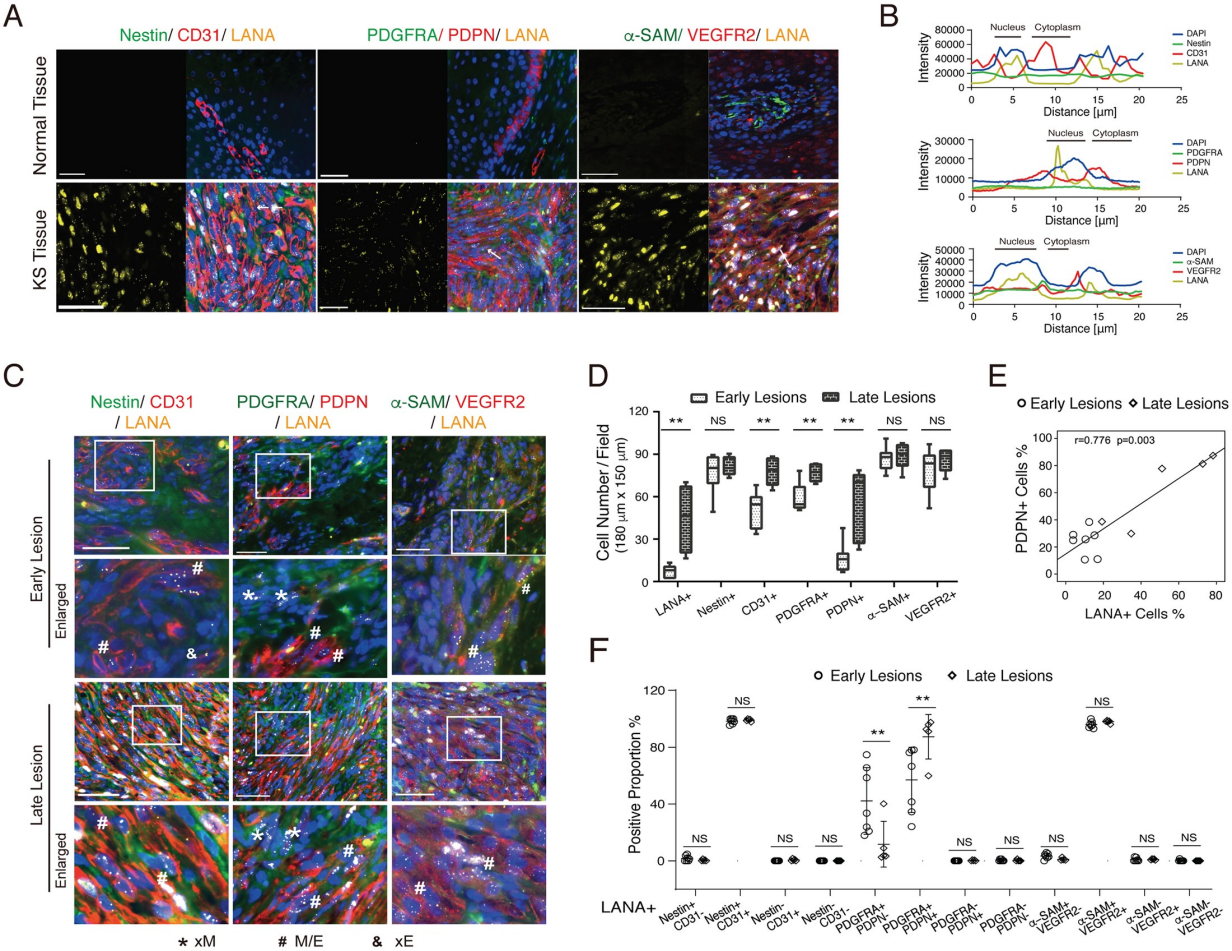
Co-expression of mesenchymal and endothelial markers in KS tissue. **(A)** Representative immunofluorescence images of AIDS-KS lesion tissues (lower) and their adjacent normal skin tissues (upper) stained with antibodies against mesenchymal (Nestin, PDGFRA, or α-SAM in green), endothelial (PDPN, CD31 or VEGFR2 in red), and KSHV (LANA in yellow) markers. The nuclei were counterstained with Hoechst 33342 (blue). Scale bars, 50 μm. Images of each individual channel for mesenchymal, endothelial and KSHV marker, respectively, are shown in S1 Fig. (**B)** Triple labeling for mesenchymal, endothelial, and KSHV markers, demonstrating the colocalization of these three fluorescent signals in the same KS tumor cell, as revealed by the plot of fluorescence intensity profiles across a white arrow in panel A. **(C)** Co-expression of mesenchymal (Nestin, PDGFRA, and α-SAM), endothelial (PDPN, CD31, and VEGFR2), and KSHV (LANA) markers in KS early (patch and plaque) and late lesions (nodular). Boxed areas are enlarged. Scale bar, 50 μm. (**D)** Number of LANA, Nestin, CD31, PDGFRA, PDPN, α-SAM, and VEGFR2 positive cells was counted from 4–6 individual fields (180μm x 150μm) composed mostly of spindle tumor cells and vessels in KS early (n = 7 samples) or late lesions (n = 5 samples). Numbers were compared by Chi-2 test. (**E)** Spearman’s test shows a correlation between LANA expression and cells positive for PDPN in early and late KS tumors. (**F)** Percentage of Nestin-/CD31-, Nestin+/CD31-, Nestin+/CD31+, Nestin-/CD31+ and PDGFRA-/PDPN-, PDGFRA+/PDPN-, PDGFRA+/PDPN+, PDGFRA-/PDPN+, and α-SAM-/VEGFR2-, α-SAM+/VEGFR2-, α-SAM+/VEGFR2+, α-SAM-/VEGFR2+ cells in LANA+ spindle tumor cells in early or late KS lesions.

KS tumors express heterogeneous markers characteristic of many cell lineages, including endothelial markers and mesenchymal stem cell markers [11,28–30]. We wondered if the unique feature of KS truly reflects the simultaneous presence of different lineage markers on the same tumor cell rather than the presence of distinct subpopulations in different differentiation statuses. Toward this question, we performed a triple immunofluorescence assay on KS clinic samples for a mesenchymal marker (Nestin, PDGFRA, or α-SAM), an endothelial marker (PDPN, CD31, or VEGFR2), and a KSHV marker (LANA). Samples from twelve AIDS patients, including early (macules/papules, n = 7) and late KS lesions (nodules, n = 5), were analyzed. We observed that LANA-positive spindle-shaped cells mostly expressed both mesenchymal stem cell markers (Nestin, PDGFRA, or α-SAM) and endothelial markers (PDPN, CD31, or VEGFR2) simultaneously. In contrast, LANA-negative cells did not express endothelial markers PDPN, CD31, and VEGFR2 but only mesenchymal stem cell markers PDGFRA, Nestin, and α-SAM (Fig 1A and S1 Fig). The co-expression pattern of these proteins was confirmed by plotting the fluorescence intensity across a line using ZEN profile tools as displayed in Fig 1B. Furthermore, the numbers or proportions of LANA+ PDPN+, LANA+CD31+, and LANA+VEGFR2+ cells were found significantly higher in the late KS lesions than in the early lesions (Fig 1C and 1D). The percentage of PDPN-positive cells correlates with the percentage of LANA-positive cells (Fig 1E). Almost all LANA-positive spindle cells expressed Nestin, CD31, α-SAM, and VEGFR2 regardless of early or late KS stages; the proportions of LANA+ PDGFRA+ PDPN+ cells increase with KS progression (Fig 1F and S1 Fig). These results indicate that (i) KS lesions contained a large number of tumor cells with a hybrid mesenchymal/endothelial (M/E) state that may result from an incomplete mesenchymal-to-endothelial transition of MSCs; (ii) the M/E state is strongly associated with the presence of KSHV in the tumor cells; (iii) the proportion of KSHV-positive M/E hybrid cells increases with KS progression.

**Fig 2:**
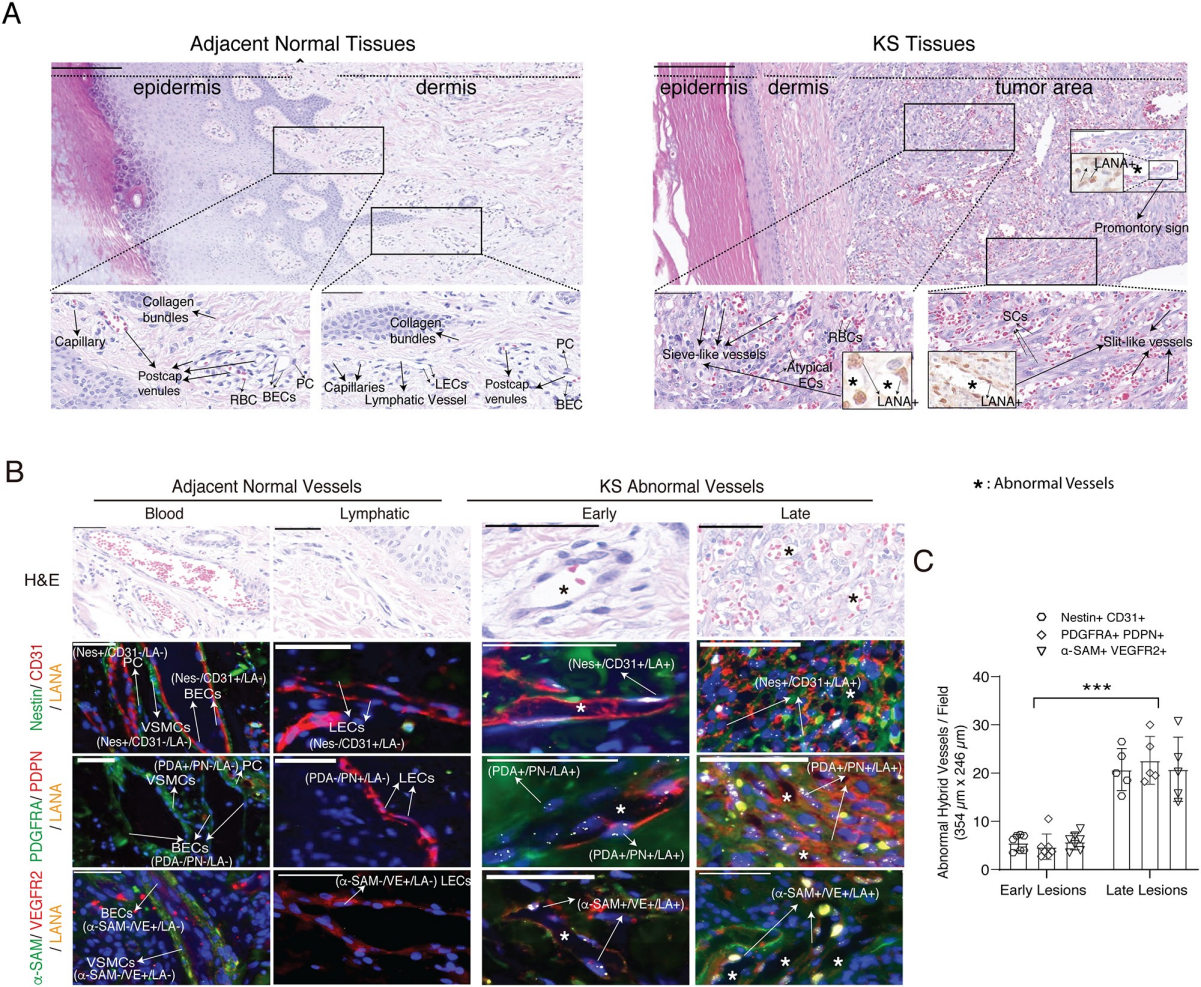
Kaposi’s sarcoma abnormal vessels are made of lined LANA-positive cells with mesenchymal and endothelial markers. **(A)** The difference in morphology and structure between normal vessels (left) and KS specialized vessels (right). Higher magnifications of the black-boxed areas are shown underneath. Scale bar, 200 μm (upper), 50 μm (lower). Asterisk, KS abnormal vessels. **(B)** Expression patterns of mesenchymal and endothelial markers in normal and KS abnormal vessels. Samples were stained with antibodies against Nestin/PDGFRA/α-SAM (green), CD31/PDPN /VEGFR2 (red), and LANA (yellow), and the nuclei were counterstained with Hoechst 33342 (blue). Scale bar, 50 μm. Asterisk, KS abnormal vessels. **(C)** LANA+ abnormal hybrid vascular density in early and late KS tumors. The number of vessels was quantified from 4–6 individual fields (354μm x 246μm) for each KS tumor sample. Error bars represent mean ± SEM. All statistical analyses were performed using the Mann-Whitney U test. *p < 0.05, **p < 0.01, ***p < 0.001, NS, not significant.

One of the most notable characteristics in KS lesions is abundant neovascularity, which results in the proliferation of irregular, jagged vascular channels accompanied by erythrocyte diapedesis. KS abnormal vessels differ from their normal counterpart, displaying unique features characterized by slit-like and sieve-like morphology. The tumor vascular channels surround and protrude into native vessels resulting in characteristic promontory signs (Fig 2A). To characterize the KS specialized vessels, we sought to determine the cellular origin of KS vessels by analyzing the above triple immunohistochemistry images for specific marker profiles of the vessels. In the KS adjacent normal blood and lymphatic vessels, vascular endothelial cells are CD31+, VEGFR2+, PDPN-, PDGFRA-, α-SAM- and Nestin-; lymphatic endothelial cells are CD31+, VEGFR2+, PDPN+, PDGFRA-, α-SAM- and Nestin-, whereas vascular smooth muscle cells and pericytes are PDGFRA+, Nestin+, α-SAM+ CD31-, PDPN-, VEGFR2- in both vessels (Fig 2B). This observation is consistent with the nature of normal blood vessels that are composed of blood endothelial cells (BECs) and vascular smooth muscle cells (VSMCs), and lymphatic vessels that are lined by lymphatic endothelial cells (LECs) and a thin layer of smooth muscle cells. In contrast, KS abnormal vessels were made of lined LANA-positive cells that were PDGFRA+, PDPN+ or PDGFRA+, PDPN- when stained with PDGFRA and PDPN antibodies; Nestin+, CD31+ when staining with Nestin and CD31 antibodies, and α-SAM+, VEGFR2+ when staining with α-SAM and VEGFR2 antibodies, suggesting that KS aberrant vessels are formed by KS tumor cells with hybrid M/E and xM phenotypes (Fig 2B). Moreover, the density of LANA+ abnormal hybrid vessels increases with KS progression (Fig 2C). Therefore, KS specialized irregular jagged vessels were lined by LANA+ spindle tumor cells rather than normal endothelial cells and these vessels are prone to leakage of fluid and extravasation of RBCs. Taken together, these results indicate that KS spindle cells display a hybrid M/E state through MEndT and KS abnormal vessels derive from KSHV-infected MSCs.

### KSHV infection induces MSC differentiation into mesenchymal/endothelial hybrid state cells through MEndT *in vitro*

**Fig 3:**
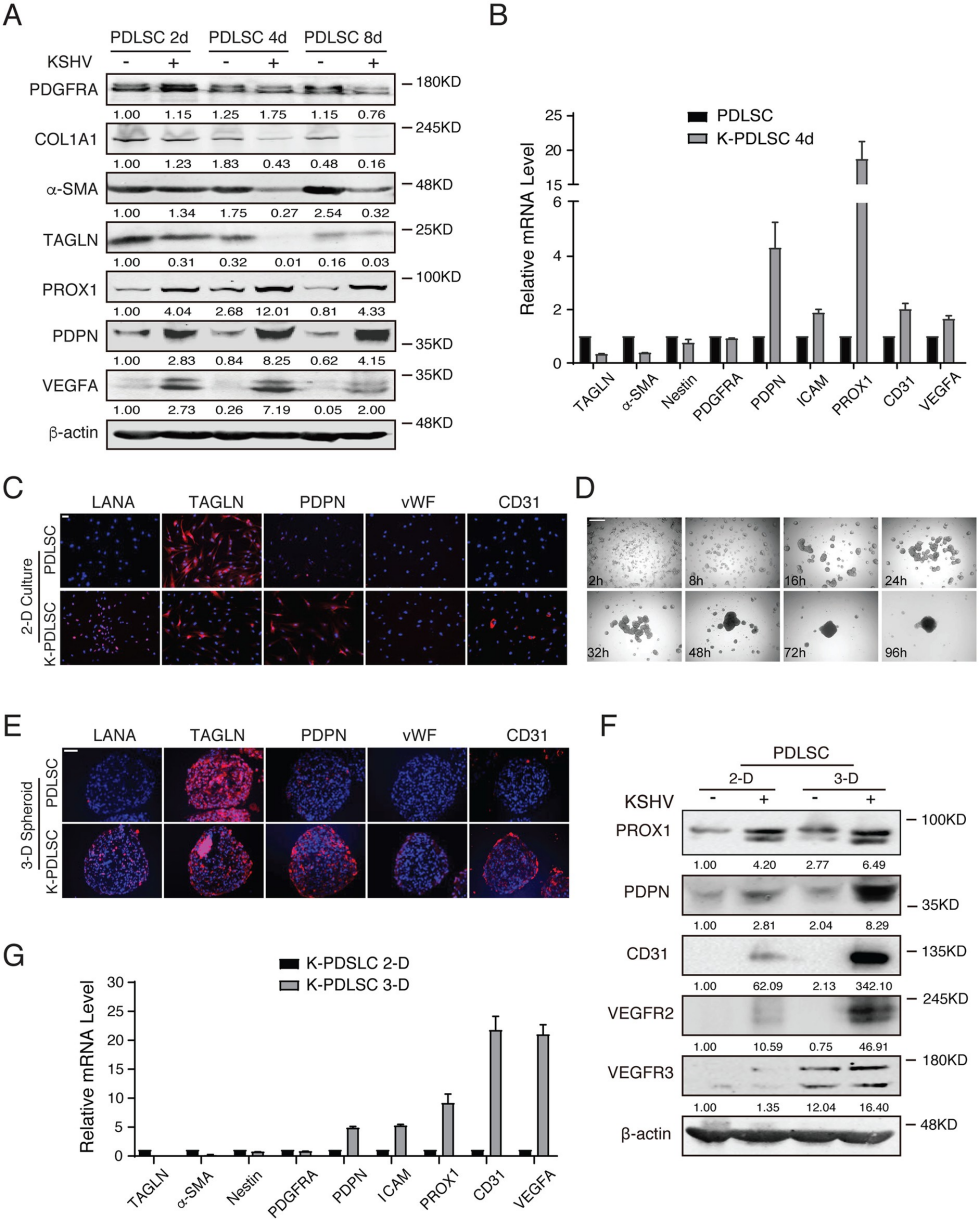
KSHV infection initiates a differentiation process converting MSCs from mesenchymal phenotype to M/E hybrid state. **(A)** Cell lysates from mock- and KSHV-infected PDLSCs at indicated time points were immunoblotted for PDGFRA, COL1A1, α-SAM, TAGLN, PROX1, PDPN, VEGFA, and β-actin. (**B)** Relative mRNA levels of mesenchymal and endothelial related genes in mock- and KSHV-infected PDLSCs (K-PDLSCs) after 4 days infection. (**C)** Mock- and KSHV-infected PDLSCs (2-D) were immunostained for LANA, TAGLN, PDPN, vWF, and CD31 at 4 days post-infection. Scale bar, 50 μm. (**D)** A time course of K-PDLSC aggregating to form spheroid in non-adherent plates. Scale bar, 500 μm. (**E)** Expression of LANA, TAGLN, PDPN, vWF, and CD31 in mock- and KSHV-infected PDLSC spheroid (3-D) at 4 days post-infection. Scale bar, 50 μm. (**F)** The expression of endothelial markers in mock- and KSHV-infected PDLSCs under 2-D or 3D cell culture for 4d. (**G)** The mRNA expression level of *TAGLN*, *α-SAM*, *Nestin*, *PDGFRA*, *PDPN*, *ICAM*, *PROX1*, *CD31*, and *VEGFA* was analyzed by RT- qPCR in K-PDLSC spheroids in comparison with their parallel 2D culture.

The triple immunofluorescence assay of KS lesions revealed the hybrid M/E phenotype of KSHV-positive spindle-shaped tumor cells. This observation compelled us to hypothesize that KS spindle cells arose from mesenchymal stem cells and KSHV initiates an MEndT process converting cells from mesenchymal phenotype to hybrid M/E phenotype. To prove this hypothesis, we attempted to reproduce this process in cultured mesenchymal stem cells to investigate whether KSHV infection could induce reprogramming of MSCs, leading to endothelial-like or M/E hybrid cells and abnormal angiogenesis as observed in KS lesions. First, PDLSCs were grown in 2-D culture and infected with KSHV. The changes of the cells in mesenchymal and endothelial cell markers were examined at different time points using Western blot, RT-qPCR, and immunofluorescence assay (IFA). The result showed that some mesenchymal markers, such as COL1A1, α-SAM, and TAGLN, faded and endothelial markers PROX1 and PDPN increased starting at the fourth day post-infection (Fig 3A). In consistent with KS lesions, mesenchymal stem cell markers PDGFRA and Nestin remained unchanged after KSHV infection (Fig 3B). However, KSHV infection did not result in the substantial expression of endothelial markers CD31 and vWF, which are expressed in KS (Fig 3C), suggesting that the 2-D cell culture system may not faithfully represent the MEndT in tumors.

Three-dimensional (3-D) organotypic cultures allow the mimic function of living tissue and probably provide information encoded in tissue architecture. The 3-D culture was used in mesenchymal stem cells in that MSC spheroids display enhanced differentiation capability compared to 2-D culture [31,32]. We established a 3-D spheroid model by seeding mock- and KSHV-infected PDLSCs in a low attachment condition. As time went by, PDLSCs formed a decentralized network, and then numerous small aggregates progressively assembled into a single central spheroid (Fig 3D). Once aggregated, the spheroid did not change in size but was generally compacted. The expression spectrum of mesenchymal and endothelial cell markers in mock- and KSHV-infected PDLSC spheroids was examined by IFA. As shown in Fig 3E, the endothelial markers PDPN and CD31were induced, and mesenchymal marker TAGLN was decreased in KSHV-PDLSC spheroids compared with control spheroids. But PDGFRA expression remained unchanged between mock- and KSHV-infected PDKSC spheroids. The mesenchymal/endothelial marker profiles in 2-D culture and 3-D spheroids of KSHV-PDLSCs were compared using Western blot and RT-qPCR. Results showed that endothelial markers PROX1, PDPN, CD31, VEGFR2, and VEGFR3 increased, and the transcription of endothelial marker genes *PDPN*, *ICAM*, *PROX1*, *CD31*, and *VEGFA* were dramatically up-regulated in 3-D spheroids, confirming the occurrence of MEndT in KSHV-PDLSC spheroids (Fig 3F and 3G). Overall, the 3-D organotypic culture provides a suitable environment allowing the differentiation of KSHV-infected MSCs into hybrid M/E cells and demonstrating that KSHV infection of MSCs sufficiently induces the MEndT (or incomplete MEndT) process, generating hybrid M/E cells closely resembling the spindle cells in KS lesions.

### KSHV-induced MEndT leads to incomplete endothelial differentiation

**Fig 4:**
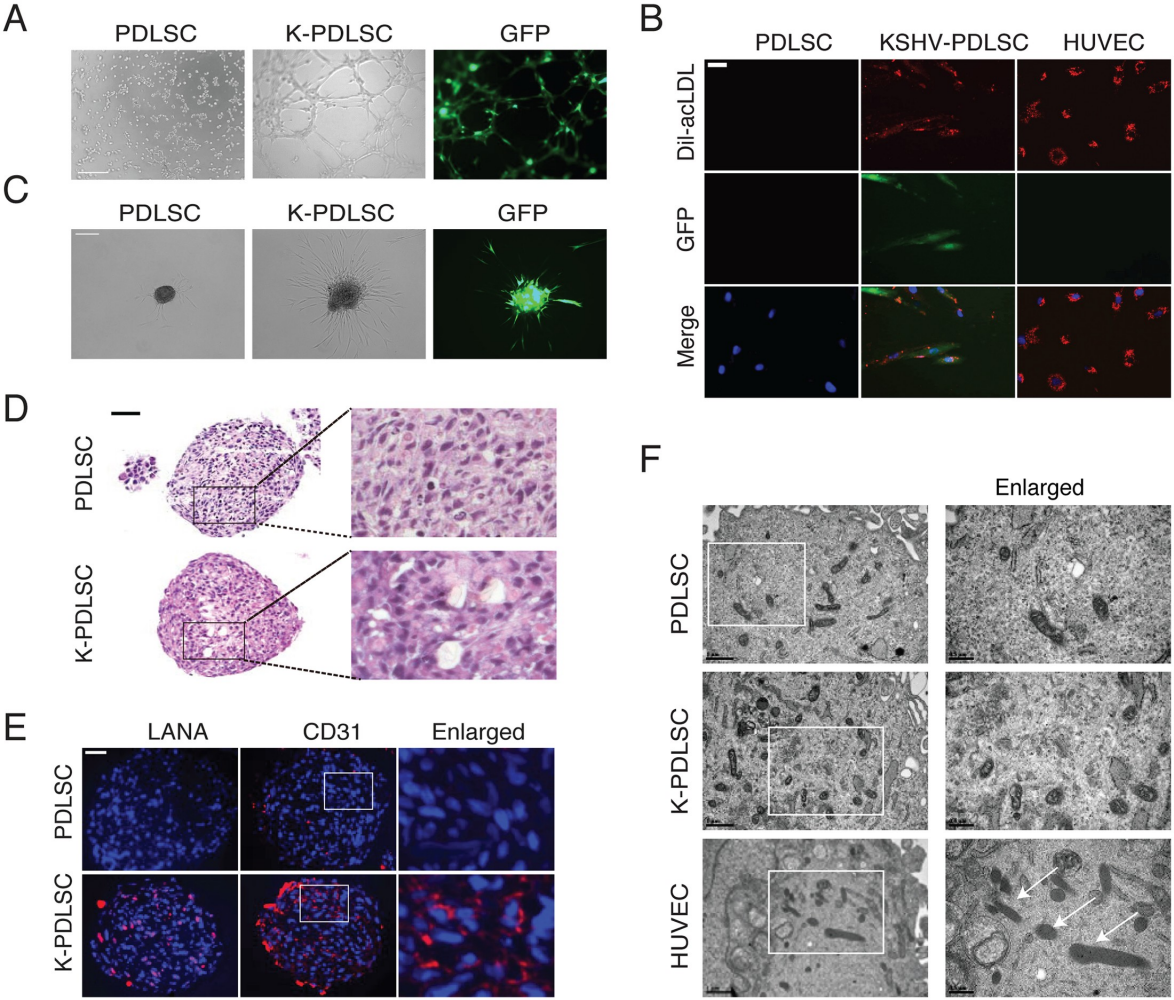
The hybrid M/E state is enriched through KSHV-mediated MEndT and displays partial endothelial characteristics. **(A)** Tubule formation assays were performed with mock- and KSHV-infected PDLSCs (K-PDLSCs). Scale bar, 500 μm. (**B)** Mock- and KSHV-infected PDLSCs were incubated with DiI-acLDL for 4 hours. DiI-acLDL uptake (red), as well as KSHV-GFP infection (green), were analyzed with a fluorescence microscope. Scale bar, 100 μm. (**C)** Mock- and KSHV-infected PDLSC spheroids were embedded into Matrigel, and the sprouting length was analyzed by a Zeiss fluorescence microscope. Scale bar, 200 μm. (**D)** H&E staining of PDLSC and K-PDLSC spheroid sections. Scale bar, 50 μm. (**E)** The expression of CD31 in the spheroids of PDLSC and K-PDLSC was detected by IFA. Scale bar, 50 μm. (**F)** Mock- and KSHV-infected PDLSCs, along with HUVECs, were examined for Weibel–Palade bodies (WPBs) under a transmission electron microscope. Scale bar, 1 μm. Original magnification, ×2 (enlarged insets).

Then, we asked if KSHV-induced hybrid M/E state cells have acquired functional characteristics of endothelial cells in addition to expressing endothelial markers. To this end, we evaluated KSHV-infected PDLSCs for their endothelial characteristics. Matrigel tubule formation assay was carried out for the acquisition of endothelial and angiogenesis properties and showed that KSHV-infected PDLSCs exhibited increased ability to form capillary-like structures in comparison to mock-infected PDLSCs (Fig 4A). The uptake of acetylated low-density lipoprotein (Ac-LDL) is a hallmark of endothelial cells and macrophages [33]. KSHV-infected PDLSCs were found to possess an Ac-LDL uptake capacity similar to HUVECs (Fig 4B). KSHV induced a notable outgrowth in KSHV-infected PDLSC spheroids whereas rare sprouting was observed in mock-infected PDLSCs (Fig 4C). Interestingly, KSHV-infected PDLSCs spontaneously formed many vessel-like structures in the 3D spheroids, but such structures were not seen in mock-infected PDLSC spheroids (Fig 4D). Immunofluorescence staining of these lumens showed the expression of pan-endothelial marker CD31 in KSHV-infected PDLSC spheroids but not in control spheroids (Fig 4E).

Weibel–Palade bodies (WPBs) are mature endothelial cell-specialized organelles. To investigate the endothelial differentiation grade of KSHV-infected PDLSCs, we examined whether KSHV-infected PDLSCs displayed WPBs using a transmission electron microscope. Weibel–Palade bodies were not observed in both KSHV-infected PDLSCs and uninfected control PDLSCs (Fig 4F). Taken together, our findings indicate that KSHV infection can reprogram MSCs to acquire endothelial markers and functions through MEndT. However, KSHV-induced endothelial lineage differentiation is incomplete as cells retain specific mesenchymal markers and lack endothelial cell specialized organelles Weibel–Palade bodies (WPBs), exhibiting a striking resemblance to KS spindle cells [8].

### Characterization of KSHV-infected MSCs residing in the intermediate hybrid M/E state

**Fig 5:**
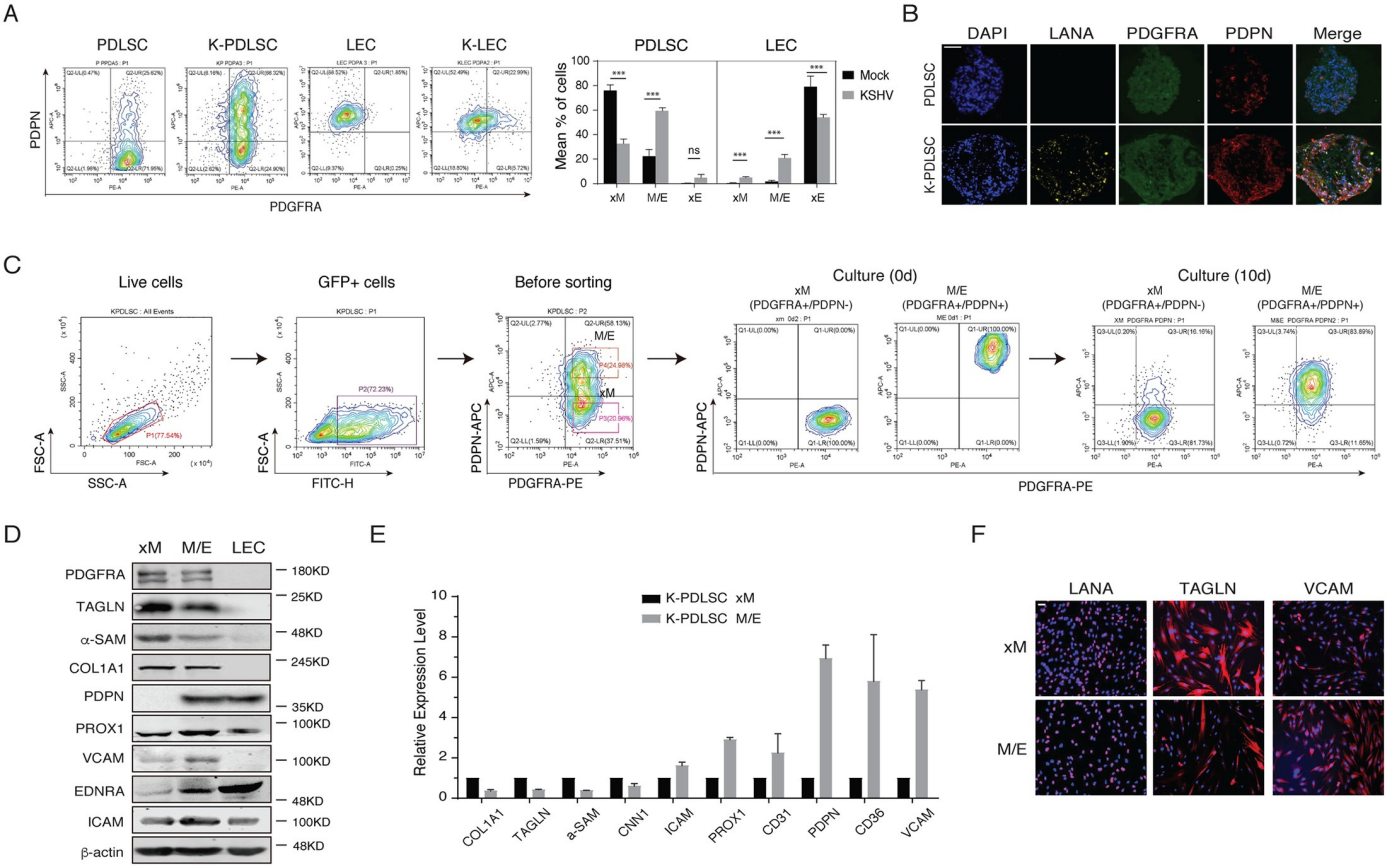
Characterization of KSHV-infected PDLSCs of distinct states in the mesenchymal-to-endothelial differentiation spectrum. **(A)** Mock- and KSHV-infected PDLSCs and LECs were examined for PDGFRA and PDPN expression profile by flow cytometry analysis. Three subpopulations (xM, hybrid M/E, and xE) were quantified based on the PDGFRA and PDPN profiles (*n* = 3 independent experiments). Statistical analyses were performed using two-tailed Student’s test and P-values were calculated by GraphPad Prism. *p < 0.05, **p < 0.01, ***p < 0.001. (**B)** PDGFRA and PDPN expression in PDLSC and K-PDLSC spheroids at 4 days post-infection were analyzed by IFA. Scale bar, 50 μm. (**C)** K-PDLSCs were stained for PDGFRA and PDPN and sorted by flow cytometry. The purified xM and M/E populations were cultured for 10 days and their PDGFRA/PDPN profiles were examined for their phenotypic plasticity. (**D)** Western blot analysis of xM, M/E, and LECs for their mesenchymal and endothelial markers. (**E)** The expression profiles of mesenchymal and endothelial markers in hybrid M/E and xM state cells were analyzed at the mRNA level by RT-qPCR. (**F)** IFA analysis of LANA, TAGLN, and VCAM in xM and M/E state cells. Scale bar, 50 μm.

To characterize KSHV-infected MSCs residing in different phenotypic states along the mesenchymal–endothelial spectrum, we used mesenchymal (PDGFRA) and lymphatic endothelial (PDPN) double markers to track the KSHV-mediated MEndT program. The PDGFRA/PDPN profiles of mock- and KSHV-infected PDLSCs were analyzed by flow cytometry (Fig 5A). Mock- and KSHV-infected LECs were included as references. PDLSCs were positive for PDGFRA but very few expressed PDPN, whereas LECs were PDGFRA-negative and PDPN-positive. KSHV infection of MSCs generated a small percentage (5%) of PDGFRA- PDPN+ (xE) cells, 60% of PDGFRA+ PDPN+ (M/E hybrid) cells and 35% of PDGFRA+ PDPN- (xM) cells. Considering the KSHV infection rate was between 70–80%, most KSHV-infected MSCs were believed to be converted to M/E and xE cells. Similarly, the KSHV-infected PDLSCs in 3-D culture spheroids generated a large number of M/E hybrid state cells and rare xE state cells (Fig 5B). On other hands, KSHV infection also induced LECs to generate PDGFRA+ PDPN+ (M/E hybrid) cells. Therefore, KSHV infection can reprogram undifferentiated MSCs and differentiated endothelial cells into M/E hybrid status.

To characterize these subpopulations, we isolated xM, hybrid M/E, and xE cells from KSHV-infected PDLSCs using PDGFRA/PDPN antigen marker combination and tracked their M and E status during propagation *in vitro*. Highly pure xM (PDGFRA+ PDPN-) and hybrid M/E (PDGFRA+ PDPN+) were collected, and the status of xM and hybrid M/E cells were found sustainable after ten days culture, indicating both xM and M/E subpopulation were low-plastic and resided stably in an intermediate phenotypic state *in vitro* (Fig 5C). These xM and M/E subpopulations were characterized for their mesenchymal and endothelial status by Western blot, RT-qPCR, and immunofluorescence (IFA) analyses (Fig 5D–5F). Hybrid M/E state cells, as expected, displayed a mixture of mesenchymal and endothelial traits, including the simultaneous expression of PDGFRA and PDPN together with other M and E markers. Some mesenchymal cytoskeletal markers (COL1A1, TAGLN, and α-SAM) were down-regulated in M/E cells compared to xM cells in mRNA and protein levels. On the other hand, levels of several endothelial markers (PROX1, VCAM, EDNRA, and ICAM) were significantly elevated in M/E cells relative to xM cells (Fig 5D–5F).

### Hybrid M/E state cells manifest tumorigenic phenotypes and the potential to form KS-like lesions after ectopic transplantation

**Fig 6:**
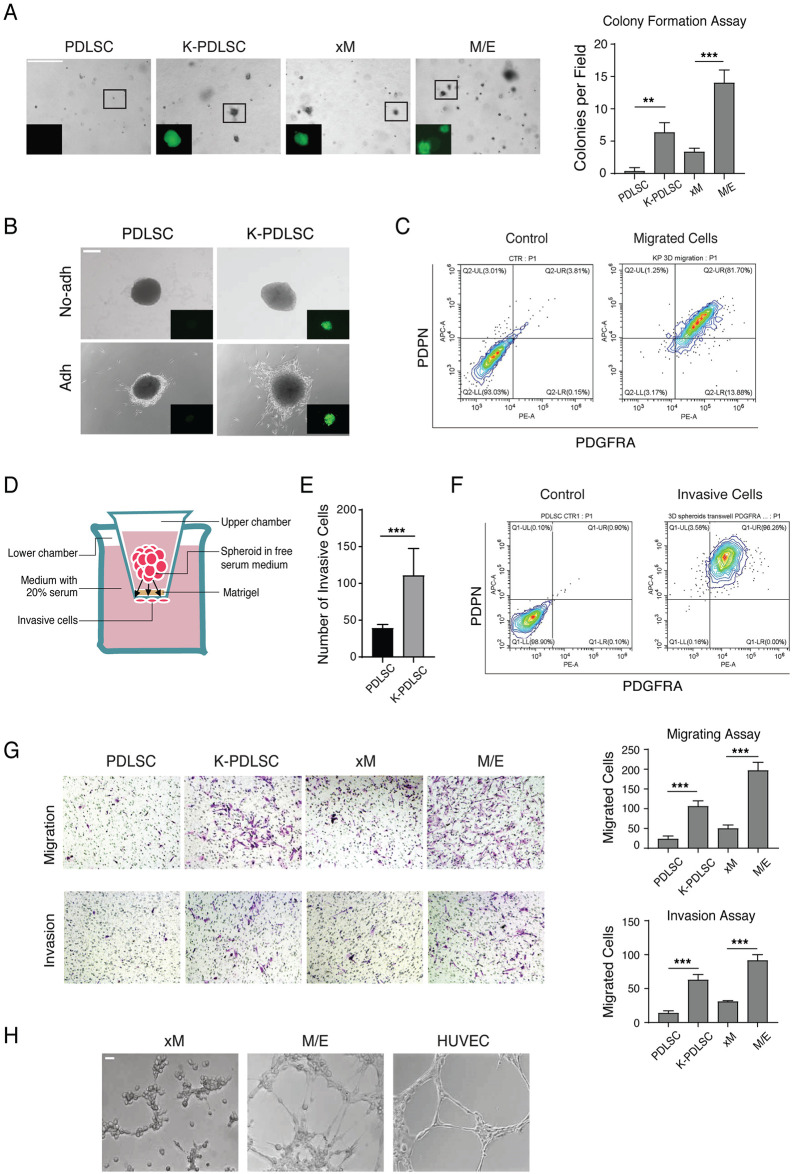
KSHV endows PDLSCs with tumorigenic Properties and M/E exhibits high tumorigenicity. **(A)** Soft agar colony formation assay to determine anchorage-independent cell growth in PDLSCs, K-PDLSCs, xM, and M/E state cells. Representative cell colonies in soft agar are shown. Scale bar, 200 μm. **(B)** Images of PDLSC and K-PDLSC spheroids 48h after transferring onto nonadherent and adherent plates. Scale bar, 200 μm. **(C)** FASC analysis of the migrated cells detached from K-PDLSC spheroids for PDGFRA/PDPN profiles. The cells that were stained with mouse and rat IgG were used as a control for creating flow cytometry gates. **(D)** Illustration of spheroids Transwell invasion assay. (**E)** Quantitation of the number of invaded cells from PDLSC and K-PDLSC spheroids. **(F)** PDPN/PDGFRA expression profiles of the invaded cells from K-PDLSC spheroids. **(G)** Transwell migration and invasion assays of PDLSC, K-PDLSC, xM, and M/E state cells. Quantitation of cell migration and invasion was shown on the right. **(H)** Tubule formation assays were performed with xM and M/E cells. HUVECs were included as a positive control. Error bars represent mean ± SEM (n = 3). Statistical analyses were performed using the two-tailed Student’s test. * p < 0.05, ** p < 0.01, *** p < 0.001.

The resemblance between KS spindle cells and the KSHV-mediated M/E state of MSCs suggests that KS originates from KSHV-infected MSCs, and the M/E state cells have acquired KS tumorigenic properties during the mesenchymal-to-endothelial transition driven by KSHV. To confirm this speculation, we examined the M/E and xM state cells for their tumorigenic potentials including malignant transformation, migration/invasion, and angiogenesis. Soft agar colony formation assay, the standard tumorigenicity test, was used to evaluate cellular anchorage-independent growth under a low-nutrient and -oxygen microenvironment [34,35]. Mock- and KSHV-infected PDLSCs, as well as xM and hybrid M/E cells isolated from KSHV-infected PDLSCs, were subjected to colony-forming assay. KSHV-infected PDLSCs produced colonies in soft agar, but mock-infected PDLSCs failed to develop any visible colonies. Hybrid M/E state cells formed more colonies than xM state cells (Fig 6A).

It was reported that KSHV infection of MSC increases its migration and invasion capability, which was proposed to be responsible for the tendency of KS occurring in injured or inflamed sites of the body [36]. To assess the migration ability of distinct phenotypic state cells, mock- and KSHV-infected PDLSC spheroids were seeded on adherent culture plates and observed for spindle-shaped cells migrating from the spheroids. We found that more KSHV-infected PDLSCs moved away from their spheroids than mock-infected spheroids. On nonadherent surfaces, no migration was observed with both mock- and KSHV-infected spheroids (Fig 6B). The migrating cells were analyzed for PDPN/PDGFRA expression profile to determine their MEndT status. Results showed that the migrating cells were mainly hybrid M/E state cells (Fig 6C). The invasion abilities of KSHV-PDLSC and M/E cells were assayed using a Transwell apparatus as illustrated in Fig 6D. Cells that permeated the Matrigel and reached the other side of the membrane were analyzed by flow cytometry. The number of migrated cells from KSHV-infected PDLSCs spheroids was significantly greater than mock-infected spheroids (Fig 6E). The invaded cells were almost entirely hybrid M/E state cells (Fig 6F). In addition, we compared migration and invasion properties of M/E hybrid and xM state cells using Transwell assays. The result showed that KSHV infection enhanced PDLSC’s migration and invasion ability, and hybrid M/E state cells have a higher capacity to migrate and invade than xM state cells (Fig 6G). Furthermore, angiogenic capabilities of hybrid M/E and xM were also analyzed using a Matrigel tubulogenesis assay, and hybrid M/E cells exhibited a higher ability to form capillary-like structures in Matrigel stroma than xM cells (Fig 6H).

**Fig 7:**
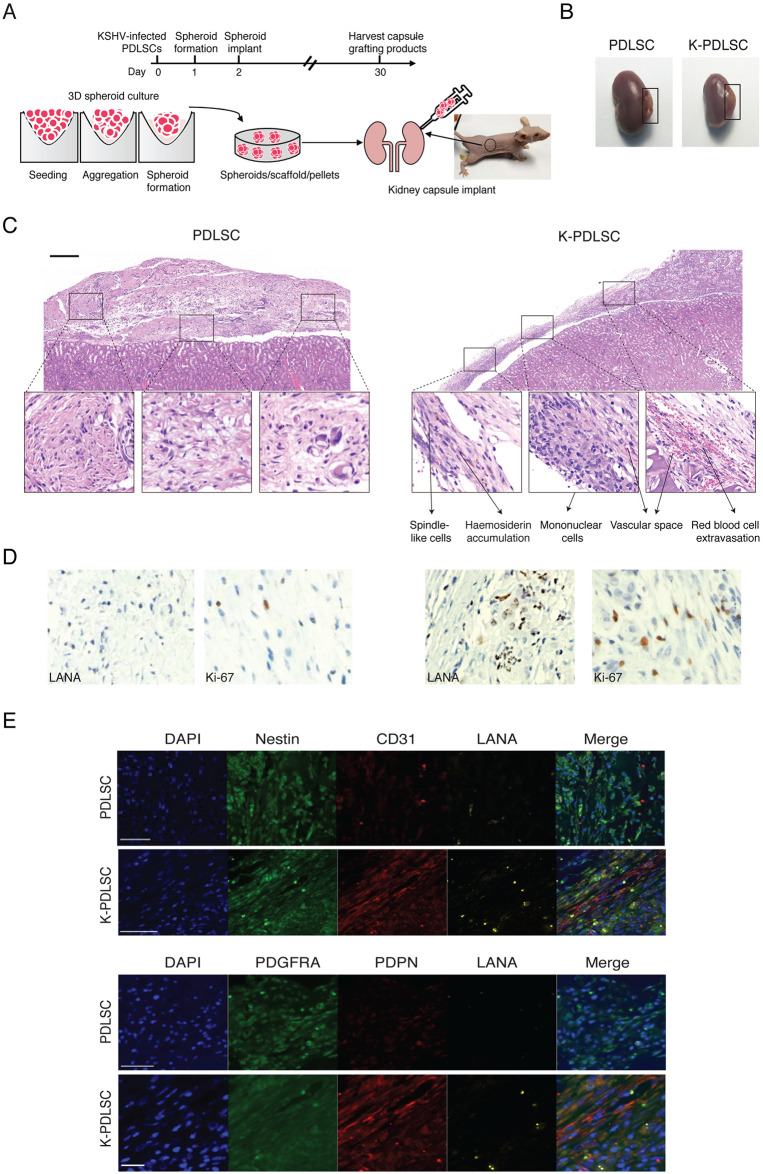
Ectopic transplantation of PDLSC and K-PDLSC spheroids in mice under the kidney capsule. **(A)** Schematic diagram illustrating the process of transplanting PDLSC and K-PDLSC spheroids under the renal capsule of nude mice. (**B)** The kidneys transplanted with PDLSC and K-PDLSC spheroids were harvested after 28 days. (*n* = 3–4 mice for each group). (**C)** Representative images of H&E staining of PDLSC and K-PDLSC spheroids transplants. Scale bar, 200 μm. **(D)** IHC staining of PDLSC and K-PDLSC spheroids transplants for LANA and Ki-67. **(E)** Immunofluorescent overview of Nestin, CD31, PDGFRA, PDPN, and LANA expression in PDLSC and K-PDLSC spheroids under the renal capsule. Scale bar, 50 μm.

Then we evaluated the capability of KSHV-infected PDLSCs in forming KS-like tumors *in vivo* using a 3D spheroids ectopic transplantation model (Fig 7A). Mock- or KSHV-infected PDLSC spheroids embedded within the scaffold were transplanted under the kidney capsule of immunocompromised mice. Kidneys were harvested after four weeks for immunohistochemical analysis (Fig 7B). Hematoxylin and eosin staining showed spindle-shaped cells, abundant new vessel networks, slit-like vascular spaces with leaky erythrocytes, haemosiderin accumulation, and mononuclear cell infiltrates in the region of inoculation of KSHV-infected MSC spheroids, resembling to these seen in Kaposi’s sarcoma lesion. In contrast, these tumor properties were not seen in mock-infected MSC spheroids transplants (Fig 7C). The sections were stained with antibodies against KSHV antigen LANA and proliferation marker Ki-67 and results showed that the KSHV-infected graft spindle-shaped cells expressed LANA and Ki-67 (Fig 7D, right).

The mock- and KSHV-infected PDLSC implants were subjected to triple IFA for mesenchymal and endothelial markers with antibodies against LANA, PDGFRA, PDPN or LANA, Nestin, CD31. The result showed that cells in KSHV-infected PDLSC implants co-expressed PDGFRA and PDPN, as well as Nestin and CD31, while mock-infected implants expressed very low levels of PDPN and CD31 (Fig 7E) The triple IFA results closely resemble those observed in KS lesions (Fig 1A) and suggest that KSHV-infected MSCs can be transformed into KS-like cells through MEndT.

### vFLIP plays a crucial role in the acquisition of hybrid M/E state and tumorigenesis

**Fig 8:**
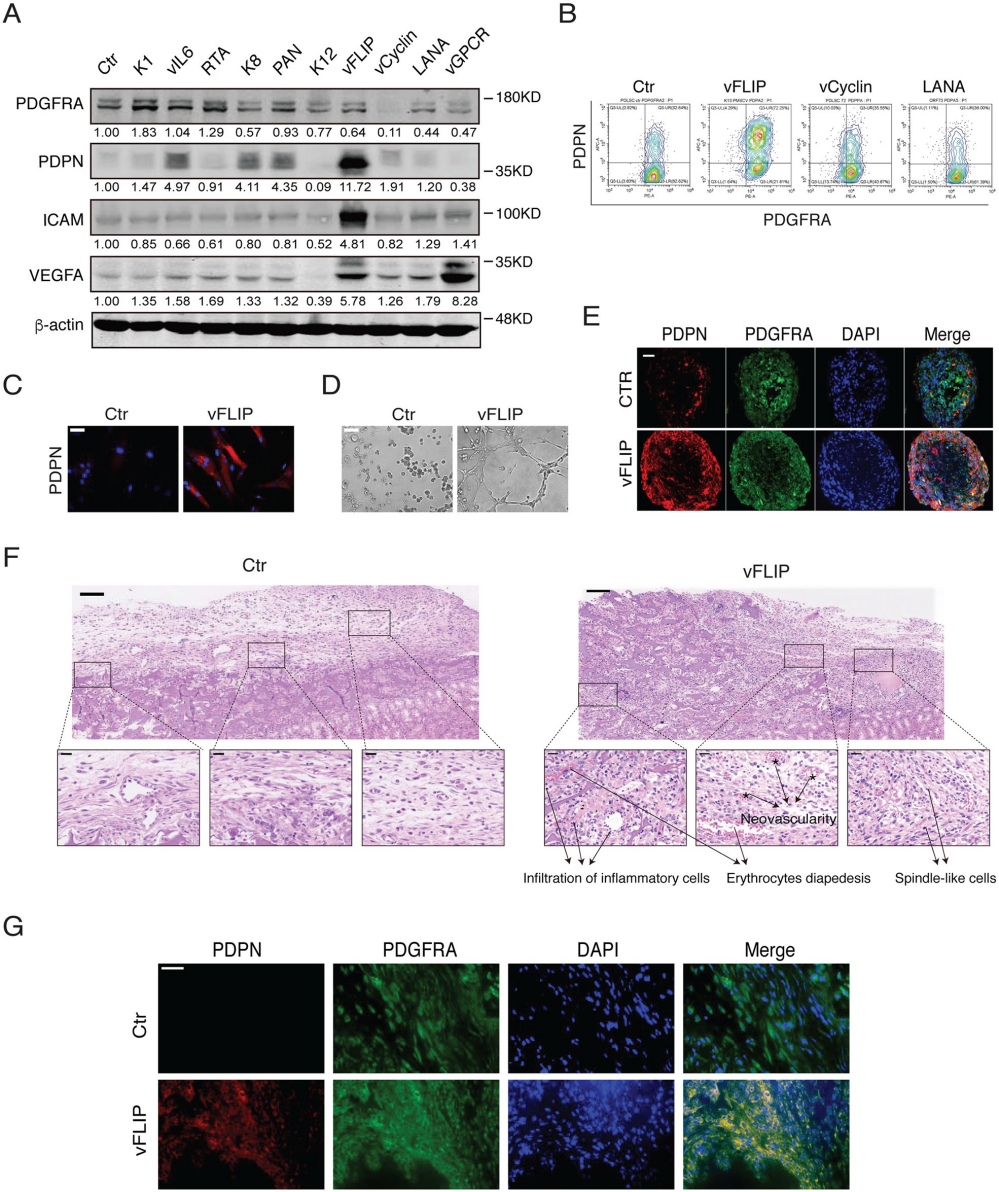
vFLIP promotes MEndT of MSCs and enhances the formation of hybrid M/E state cells and tumorigenesis. (A) PDLSCs were transduced with different viral gene expression vectors as indicated. The expression of some of these viral genes in cells was verified by Western analysis (S2 Fig). The effects of these viral gene products on the expression of PDGFRA, PDPN, ICAM, and VEGFA in PDLSCs were analyzed by Western blotting. **(B)** vFLIP-, vCyclin- and LANA-expressing PDLSCs were examined for hybrid M/E state cells using FASC. (**C)** Immunofluorescence staining of vFLIP-expressing PDLSCs for PDPN expression. Scale bar, 20 μm. (**D)** vFLIP-expressing PDLSCs were subjected to a tubule formation assay. Scale bar, 200 μm. (**E)** vFLIP-expressing PDLSC spheroids were examined by IFA for PDGFRA/PDPN expression profiles. Scale bar, 50 μm. **(F)** Representative images of H&E staining of PDLSC and vFLIP-PDLSC spheroids transplanted in mice under the kidney capsule. Scale bar, 100 μm (upper) and 20 μm (lower). (*n* = 3–4 mice for each group). **(G)** Immunofluorescent overview of PDGFRA/PDPN expression in PDLSC and vFLIP-PDLSC spheroids under the renal capsule. Scale bar, 50 μm.

To understand how KSHV promotes MEndT and what viral gene products involves in the process to generate M/E state cells, a class of viral genes, including K1, vIL-6, RTA, K8, PAN, K12, vFLIP, v-Cyclin, LANA, and vGPCR, were ectopically expressed in PDLSCs by transducing their lentiviral vectors. Among these viral genes, vFLIP was found to be able to increase the expression of endothelial markers PDPN, ICAM, and VEGFA by 5- to 12-fold (Fig 8A). The expression of vFLIP in PDLSCs also notably increased the proportion of hybrid M/E state (PDGFRA+ /PDPN+) cells (Fig 8B) and the upregulation of PDPN in vFLIP expressing cells in comparison to control PDLSCs were revealed by immunofluorescence staining (Fig 8C). Furthermore, vFLIP expression enhanced the angiogenesis of PDLSCs (Fig 8D). In 3D organotypic cultures, vFLIP-expressing PDLSCs spheroids generated a large number of hybrid M/E state cells (Fig 8E). vFLIP-expressing PDLSCs and control spheroids were transplanted into nude mice under the kidney capsule. Histopathologic examination of hematoxylin and eosin-stained slides showed spindle-shaped cells, microvessels containing red blood cells, and infiltration of the inflammatory cells in the grafting region of vFLIP-spheroids rather than control spheroids (Fig 8F). Besides, vFLIP- expressing PDLSC and control implants were stained for PDGFRA and PDPN, and results showed vFLIP-expressing PDLSC implants co-expressed PDGFRA and PDPN, while control implants did not express PDPN (Fig 8G).

**Fig 9:**
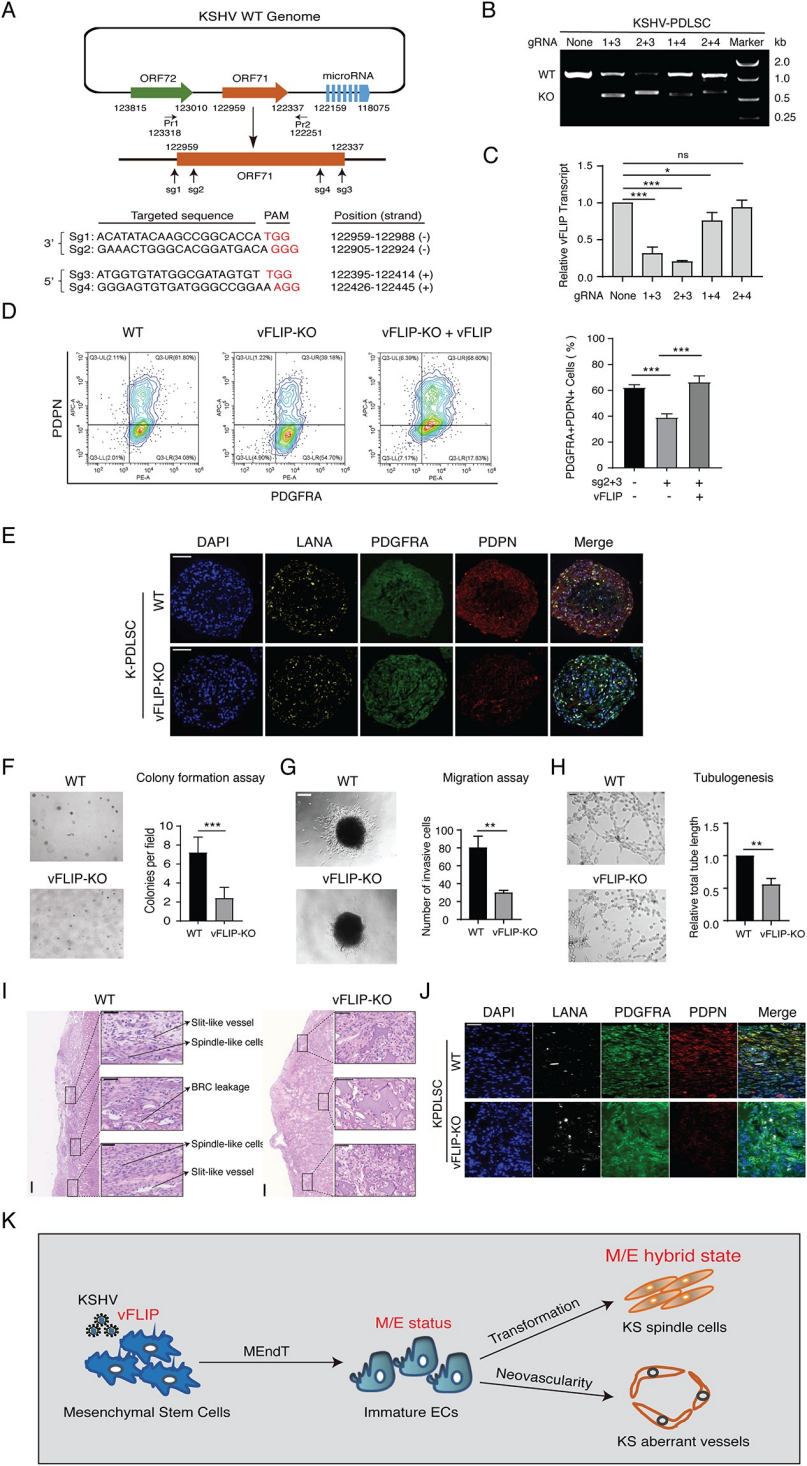
Silencing of vFLIP expression results in the abolishment of KSHV-induced MEndT and tumorigenesis. (**A)** Schematic diagram depicting the CRISPR/Cas9 editing sites in the KSHV genome. (**B)** The CRISPR/Cas9-mediated knockout efficiency of ORF71 (vFLIP) gene from the KSHV genome in KSHV-infected PDLSCs was verified by PCR with primers Pr1 and Pr2. **(C)** The vFLIP expression in ORF71 knockout cells was determined by RT-qPCR. (**D)** The effect of vFLIP knockout (vFLIP-KO) on the generation of hybrid M/E state cells, as well as the restoration of vFLIP function by ectopic expression of sgRNA-resistant vFLIP gene (S5 Fig), was quantitated by flow cytometry. (**E)** The effect of vFLIP-KO on the expression of PDGFRA and PDPN was examined with K-PDLSC spheroids by IFA. Scale bar, 50 μm. **(F)** The effect of vFLIP-KO on malignant transformation ability was examined using colony formation assay. Scale bar, 200 μm. (**G**) Images of WT and vFLIP-KO KSKV-infected PDLSC spheroids showing the effect of vFLIP-KO on cell migration capability. Scale bar, 200 μm. **(H)** The effect of vFLIP-KO on the angiogenic ability of K-PDLSCs was assayed by tubule-formation assay. Scale bar, 200 μm. **(I)** Representative images of H&E staining of WT and vFLIP-KO KSHV-infected PDLSC spheroid transplants in mice under the kidney capsule. Scale bar, 200 μm. Higher magnifications of the black boxes are shown on the right. Scale bar, 50 μm. (*n* = 3–4 mice for each group). **(J)** Immunofluorescence assay of WT and vFLIP-KO KSHV-infected PDLSC transplants for LANA, PDGFRA, and PDPN. Scale bar, 50 μm. **(K)** Schematic model for the role of KSHV vFLIP in promoting MEndT to generate hybrid M/E state cells for KS tumorigenesis and aberrant angiogenesis. Error bars represent mean ± SEM. n = 3, unless otherwise indicated. Statistical analyses were performed using the two-tailed Student’s test. *p < 0.05, **p < 0.01, ***p < 0.001.

A "loss-of-function" approach was used to verify the contribution of vFLIP to initiating MEndT and generating hybrid M/E status. We attempted to silence the expression of vFLIP using a CRISPR/Cas9-mediated gene knockout approach [37]. A series of paired guide RNAs (gRNAs) were designed targeting the flanking regions of the ORF71 gene to create ORF71 knockout (KO) (Fig 9A). Satisfied knockout efficiency was achieved with gRNAs 2 and 3, which removed ORF71 from the KSHV genome in KSHV-infected PDLSCs (Fig 9B and 9C), and the ORF71 deletion junction was verified by DNA sequencing (S3 Fig). The ORF71-KO KSHV was analyzed for its MEndT capability in PDLSCs. We found that PDGFRA+PDPN+ cells were notably reduced in vFLIP-KO cells, but complementation of vFLIP-KO mutant with a vFLIP expression vector restored the number of M/E hybrid state cells (Fig 9D). This suggested that vFLIP-KO prevented KSHV from inducing the MEndT process. In 3D organotypic cultures, the expression of PDPN and the generation of M/E state were significantly reduced in vFLIP-KO spheroids in comparison to wt KSHV-PDLSC spheroid, but PDPN expression and M/E state can be restored by complementation with a vFLIP expression vector (Fig 9E). Then we examined if vFLIP-KO affects the oncogenic properties of KSHV-infected PDLSCs, including malignant transformation, migration/invasion, and angiogenesis properties. Colony-forming assays were performed and results showed a significant reduction in the number of colonies from vFLIP-KO KSHV-infected PDLSCs compared with wild-type KSHV-infected PDLSCs (Fig 9F). 3D migration assay showed that vFLIP knockout resulted in fewer cells migrating away from KSHV-PDLSC spheroids (Fig 9G). A tubule formation assay showed decreased angiogenesis with vFLIP-KO KSHV-infected PDLSCs (Fig 9H).

The contribution of vFLIP in promoting MEndT and tumorigenesis was evaluated *in vivo* using a mice kidney capsule implant model. vFLIP-KO and wild-type KSHV-infected PDLSC spheroids were transplanted into the kidney capsule of nude mice. The transplants were harvested four weeks post-transplantation and analyzed by Hematoxylin and eosin staining. The result showed spindle-shaped cells, slit-like vessels containing red blood cells, and erythrocytes diapedesis in the implants of wild-type KSHV-infected PDLSC spheroids, while these tumor properties were not seen in vFLIP- knockout KSHV-infected PDLSC spheroids transplants (Fig 9I). The sections were subjected to triple IFA with antibodies against LANA, PDGFRA, and PDPN. Results showed that knockout of vFLIP completely abolished KSHV-induced MEndT and the generation of hybrid M/E state cells *in vivo* (Fig 9J). Taken together, these results indicated that KSHV-encoded vFLIP is crucial in the generation of M/E hybrid state cells and tumorigenesis via MEndT in MSCs.

## Discussion

Prior studies suggested that KS may arise from KSHV-infected mesenchymal stem cells (MSCs) through an MEndT process [10–12]. In this study, we investigated the MEndT process in KSHV-infected MSCs and characterized distinct differentiation subpopulations along the mesenchymal-to-endothelial spectrum. We found that KSHV promotes incomplete endothelial differentiation of MSCs to generate mesenchymal/endothelial (M/E) hybrid state cells and the hybrid M/E cells acquired oncogenic properties including generation of spindle-shaped tumor cells and aberrant neovascularity *in vitro* and *in vivo*. This finding faithfully recapitulates Kaposi’s sarcoma where proliferating KS spindle cells and the cells that line KS-specific aberrant vessels also exhibit the hybrid M/E state. Furthermore, we revealed that KSHV-encoded viral FLICE inhibitory protein (vFLIP) plays a crucial role in promoting MEndT and the generation of M/E state cells. These findings provide a new layer of evidence for KSHV-infected MSCs being the cell source of KS spindle cells and reveal novel insight into KS pathogenesis and viral tumorigenesis. A model that summarizes these findings is schematically illustrated in Fig 9K.

Kaposi’s sarcoma is a multifocal neoplasm characterized by proliferating KSHV-infected spindle-shaped tumor cells, aberrant capillaries, and infiltration of inflammatory cells. KS spindle cells exhibit extraordinarily heterogenic populations revealed by a variety of cell markers, including vascular and lymphatic endothelial, mesenchymal, smooth muscle, and hematopoietic precursor markers [38]. The origin of the spindle-shaped KS cell lineage remains contentious. Currently, the most widely accepted theory is that KS cells may derive from the endothelial cell lineage [39]. The other models for KS cell origin include that KSHV infects circulating endothelial progenitor cells [40,41] or pluripotent mesenchymal stem cells [9,42] and drives differentiation of these cells towards KS phenotypes. Recently, we and others found a series of pieces of evidence that favor the model of KS originating from mesenchymal stem cells [11,12]. Both endothelial and mesenchymal models agree that KS spindle cells reside in an intermediate phenotypic state along the mesenchymal-endothelial spectrum, which is associated with malignant phenotypes such as tumor initiation, migration, and evasion. The endothelial origin model suggests that terminally differentiated lymphatic endothelial cells can convert into KS precursors through an endothelial-to-mesenchymal transition (EndMT) [28], while the mesenchymal origin model proposes that KSHV-infected MSCs undergo a mesenchymal-to-endothelial transition (MEndT) process to acquire KS malignant phenotypes [11]. In this study, we demonstrated that KSHV infection of MSCs initiates an incomplete MEndT process and generates hybrid M/E state cells with tumorigenic properties, which recapitulates KS that comprises hybrid M/E state spindle-shaped tumor cells. The robust recapitulation of KS in our *in vitro* and *in vivo* mouse models strongly supports the hypothesis that Kaposi’s sarcoma spindle cells can arise from KSHV-infected MSCs through a mesenchymal to endothelial transition (MEndT). In particular, KSHV infection of LEC could also induce PDGFRA and α-SAM expression, suggesting a possibility that hybrid M/E KS can arise from both undifferentiated MSCs through MEndT or terminally differentiated lymphatic endothelial cells (LEC) through EndMT, of which both lead to sarcomagenesis.

One of the signature characteristics of KS is their abundant neovascularity, by which KS is clinically present as a red-purple lesion. Neovascularity in KS begins at the very early stage (patch stage) prior to the establishment of a mass. The KS-specialized new vessels are prone to leakage of fluid and extravasation of red blood cells [23]. KSHV-infected MSCs, implanted in mice under the kidney capsule, were observed to proliferate and form slit-like vascular spaces that contain erythrocytes, resembling KS abnormal vessels. More interestingly, in both kidney capsule implant and KS lesions, the abnormal vessels were found to be made of hybrid M/E state (PDGFRA+PDPN+) cells, suggesting that KSHV-infected MSC implants in mice accurately recapitulate KS in the neovascularity property. Why does KS aberrant neoangiogenesis form leaky capillaries? Our finding indicated that KS specialized irregular jagged vessels are lined by LANA positive cells with hybrid M/E phenotype derived from KSHV-infected MSCs, unlike normal blood or lymphatic vessels that are composed of blood or lymphatic endothelial cells plus a thin layer of vascular smooth muscles. Abnormal neoangiogenesis is also observed in other solid tumors. In a process termed “vascular mimicry” (VM), which has been found in breast cancer, melanoma, and nasopharyngeal carcinoma, tumors create their own, tumor cell-lined channels for fluid and blood transport [43]. In light of that both KS aberrant vessels and VM are poorly formed by tumor cells and leaking red blood cells and fluid, KS tumor cell-lined vessels can be considered as a new type of vascular mimicry and may have similar pathological roles as VM in tumor development and metastasis.

MEndT, like its reverse process EndMT, is a process of multiple and dynamic transitions and generates distinct phenotypes including mesenchymal-like (xM), endothelial-like (xE), and hybrid M/E states. KSHV-induced MEndT appears to be an incomplete differentiation process that accumulates hybrid M/E state cells as the majority of the population and allows a small percentage of cells to progress to endothelial-like (xE) state. These heterogeneities, to some degree, reflect the complex phenotypes of KS tumor cells that is not encountered in normal tissues [44]. Moreover, the hybrid M/E state cells display tumorigenic ability including tumor initiation, angiogenesis, migration, and invasiveness compared to other subpopulations, and form KS-like lesions in kidney capsule transplantation. Interestingly, as epithelial or carcinoma cells progress toward high-grade malignancy, they often activate epithelial-to-mesenchymal transition (EMT). It has been reported recently that in breast cancer and nasopharyngeal carcinoma, the cells residing in intermediate hybrid E/M (baring both epithelial and mesenchymal cell markers) state along the epithelial-to-mesenchymal spectrum exhibit the highest tumorigenicity [20,21]. Therefore, it is likely that acquisition of a hybrid E/M state, regardless of MEndT or EMT process, is essential for tumorigenicity of both carcinomas and sarcomas.

KSHV possesses two life cycles, latent and lytic. Although both latent and lytic cycles contribute to Kaposi’s sarcomagenesis [45,46], the KSHV mode of infection in KS lesions is predominantly latent and the expression of latent genes (i.e., LANA, vCyclin, and vFLIP) is sufficient in viral genomic persistence and cell transformation [47]. In this study, we found that vFLIP plays a crucial role in initiating MEndT and the generation of hybrid M/E cells. FLIP family proteins are known to be inhibitors of death receptor (DR)–induced apoptosis [48,49]. Previous studies revealed a role for vFLIP in binding to IκB kinase γ (IKKγ), thereby inducing NF-κB activation [50–52]. Furthermore, the pathogenic role of vFLIP has been explored in mice by expressing it in B cells and endothelial cells. When expressed in B cells, vFLIP was found to induce B cell transdifferentiation resulting in expansion of the macrophage/DC compartment [53]. When expressed in endothelial cells, mice developed pathological abnormalities with the appearance of elongated spindle-like cells, profound proinflammatory phenotype, and expansion of myeloid cells. Although no skin KS-like lesion was observed on these mice, this study has shown that some characteristics of KS can be induced by vFLIP [54]. Our current study showed that the expression of vFLIP in mesenchymal stem cells resulted in the acquisition of KS characteristics including spindle-shaped cells and aberrant neovascularity through initiating MEndT and generation of M/E state cells. This study revealed the mechanism of KSHV transforming MSCs to KS tumors through MEndT and demonstrated the central role of hybrid M/E state cells in KS tumorigenesis.

## Materials and methods

### Ethics statement

The collection of human samples and the use of periodontal ligament stem cells (PDLSCs) in our research were approved by the Medical Ethics Review Board of Sun Yat-sen University (approval no. 2015–028). Written informed consent was provided by study participants. The animal experiments in this study were approved by the Animal Ethics Review Board of Sun Yat-sen University (approval no. SYSU-IACUC- 2020–000317) and carried out strictly following the Guidance suggestion of caring laboratory animals, published by the Ministry of Science and Technology of the People’s Republic of China.

### Cell culture

Periodontal ligament stem cells (PDLSCs) were isolated from the periodontal ligament tissues (cells from 5 individuals were pooled to offset individual differences) and maintained in alpha minimal essential medium (α-MEM, GIBCO Life Technologies) supplemented with 10% fetal bovine serum (FBS) (GIBCO), 200 mM L-glutamine (Sigma) and antibiotics (HyClone). Human dermal lymphatic endothelial cells (LECs, ScienCell) were cultured in endothelial cell medium (ECM, ScienCell) plus supplements (ECGS, ScienCell). Human umbilical vein endothelial cells (HUVEC) were purchased from the China Center for Type Culture Collection (CCTCC, China) and cultured in endothelial cell growth medium 2 BulletKit (ScienCell). Human embryonic kidney (HEK) 293T cells (ATCC) were cultured in Dulbecco’s Modified Eagle’s Medium (DMEM) supplemented with 10% FBS and 1% penicillin/ streptomycin. All cells were cultured in a humidified 5% CO_2_ atmosphere at 37°C.

### KSHV preparation and infection

iSLK.219 cells (kindly provided by Dr. Ke Lan of Wuhan University) were induced for KSHV reactivation by treating with 1 mg/mL doxycycline and 1 mmol/L sodium butyrate for 5 days. The culture supernatants were filtered through a 0.45-μm filter and centrifuged at 100,000 g for 1 h. The rKSHV.219 pellet was resuspended in 1x PBS in 1/100 volume and stored at -80°C until use. Virus infection was carried out as previous procedure [11].

### Spheroid generation

Mock- and KSHV-infected PDLSCs were seeded in non-adherent 96-well plates pre-coated with 0.5% agarose at 15,000 to 20,000 cells per well. The spheroids were grown at 37°C for up to 4 days in a humidified atmosphere with 5% CO_2_. Spheroid generation in non-adherent plates was captured using a ZEISS microscope. To collect spheroids, the media containing the spheroids were transferred to a 15 ml conical tube, then washed twice with PBS, and centrifuged at 1000 rpm for 5 min. For histology analysis, the spheroids were collected following by agarose pre-embedding.

### Tubule formation assay

Ninety-six-well plates were coated with Matrigel (BD Biosciences, 354234) and incubated at 37°C for 30 min for solidification. Cells were plated onto Matrigel-coated wells at a density of 8 × 10^4^ cells/ well with 100 μl αMEM without FBS and incubated at 37°C with 5% CO_2_ for 8 h. The images of tube formation were captured using a ZEISS fluorescence microscope and analyzed with NIH ImageJ software.

### AcLDL uptake assay

PDLSCs were starved overnight and then cultured in the medium with 4 μg/ml Dil-acLDL (Yeasen Biotechnology, Shanghai, 20606ES76). After 4h culture, cells were fixed and analyzed under a ZEISS fluorescence microscope.

### Spheroid sprouting assay

Mock- and KSHV-infected PDLSCs were seeded in non-adherent 96-well plates pre-coated with 0.5% agarose at 2,000 cells per well. After 12–18 hours of incubation, the spheroids were harvested and mixed with Matrigel. The mixture was placed in a 48-well plate and incubated at 37°C for 1h for solidification, followed by adding medium. Three days later, the sprouting of the spheroids was observed under a ZEISS microscope and recorded.

### Flow cytometry analysis and cell sorting

Cells were detached from plates with 0.25% trypsin and washed once with 1xPBS. Then the cells were fixed with 4% paraformaldehyde for 20 min and incubated with APC-anti-human PDPN (eBioscience, 17-9381-42), and PE-anti-human PDGFRA (Sino Biological, 10556-MM02-P) for 30 min at 4°C. After washing with PBS, cells were subjected to flow cytometry analysis. Data were analyzed using CytExpert or FlowJo software.

To sort xM, M/E, and xE state cells, KSHV-infected PDLSC were trypsinized and resuspended in ice-cold 1xPBS containing 2% FBS at 1x10^7^ cells per 100 μl. PDGFRA-PE and PDPN-APC antibodies in 1:50 dilution were added into suspensions. GFP-positive cells were sorted for the first round, and PDGFRA+/PDPN- or PDGFRA+ /PDPN+ cells were isolated by 20% cutoffs for the second round of sorting. Isolated cells were allowed to culture for no more than two weeks.

### Transwell migration/invasion assay

Cell migration and invasion assays were performed using 24-well Transwell chambers with filter membranes of 12 μm pore size (Millipore Corporation, PIXP01250). Cells were detached with trypsin-EDTA, washed once with 1xPBS, and then resuspended in a serum-free medium. 3 x10^4^ cells were placed in a Transwell insert, and the medium containing 20% FBS was added to the lower chamber. After 24h incubation, non-migration cells were removed with a cotton swab. Cells that have migrated into the lower chamber were stained and counted under a ZEISS microscope. The cell invasion assay was performed with the same procedures except that Transwell inserts were pre-coated with Matrigel (BD Biosciences).

### 3-D spheroids migration assay

The spheroids were collected, washed with 1xPBS, and resuspended in a serum-free medium. 5–10 spheroids were seeded in adherent 24-well plates or nonadherent 24-well plates pre-coated with 0.5% agarose. After 48h incubation, images were captured under a ZEISS microscope. To detect the status of the migrated cell away from spheroid, the spheroids were detached with Ophthalmic forceps, and the migrated cells were collected for flow cytometric analysis after staining with PDGFRA-APC and PDPN-PE antibodies.

3-D spheroids invasion assay was performed using 24-well Transwell chambers with filter membranes of 12 μm pore size. 5–10 Spheroids resuspended in serum-free medium were seeded in an insert that has been coated with Matrigel (BD Biosciences). The lower compartment was added with a medium containing 20% FBS. Plates were then incubated at 37°C for 48h to allow cells to migrate. Non-migration cells were removed with a cotton swab. The migrated cells were stained and counted under a ZEISS microscope. The invaded cells on the lower side of the membrane were collected and analyzed by flow cytometry after staining with PDGFRA-APC and PDPN-PE antibodies.

### Soft agar colony formation

Twenty-four-well plates were coated with 0.5% agarose medium. After agar is solidified, a total of 2,000 cells, suspended in αMEM medium supplemented with 0.3% agarose and 20% FBS, were seeded onto the soft agar coated wells and incubated at 37°C in a 5% CO_2_ incubator for 3–4 weeks. Fresh culture medium was added to each well every 3 to 4 days. Colonies larger than the average size of control colonies were counted.

### Western blotting

Cell lysates were prepared as previously described [11]. Whole cell extract of 30 μg protein was resolved in SDS-PAGE and transferred onto nitrocellulose membranes. The membranes were blocked with 5% non-fat milk/PBS for 30 min and incubated with primary antibodies overnight at 4°C. The primary antibodies used in this study include anti-PDGFRA (Cell Signaling, 3174T), anti-COL1A1 (Proteintech, 67288-1-Ig), anti-ACTA2 (α-SMA, ABclonal, A7248), anti-SM22 (TAGLN, Proteintech, 10493-1-AP), anti-PROX1 (Boster, BA2390), anti-PDPN (Proteintech, 11629-1-AP), anti-VEGF-A (Immunoway, YT5108), anti-CD31 (Proteintech, 11265-1-AP), anti-VEGFR2 (Proteintech, 26415-1-AP), anti-VCAM (Proteintech, 11444-1-AP), anti-VEGFR3 (ABclonal, A5605), anti-ETAR (EDNRA, Santa Cruz, sc-135902), anti-ICAM (Proteintech, 10831-1-AP), and anti-β-actin(Sigma, A5441). Anti-IR Dye 800 or Dye 680 anti-rabbit or anti-mouse IgG antibodies (LI-COR Biosciences) were used as the secondary antibodies. An Odyssey system (LI-COR Biosciences) was used for the detection of proteins of interest.

### RT-qPCR

Total RNA was extracted with Ultrapure RNA Kit (CWBIO, CW0581). cDNA was synthesized by reverse transcription. cDNA was diluted 5 times and subjected to real-time PCR using LightCycler 480 SYBR Green I Master (Roche) with specific primers for the genes of interest. GAPDH gene was used for calibration. The stem-loop reverse transcription (RT) and quantitative real-time PCR (qRT-PCR) were carried out to examining KSHV miRNAs as described previously [55]. The primer sequences used for RT-qPCR are listed in Supporting Information S1 Table. All real-time PCR was done in triplicate.

### Kidney capsule transplantation

Spheroids were collected and washed once with 1xPBS. Approximate 100 spheroids were placed on a single 3 × 2 × 2-mm sterile gelfoam scaffold to culture for 1 day in medium. Recipient female nude mice (6–8 weeks old, n = 3–5) were weighed and anesthetized with isoflurane strictly following the animal care Guidelines. Via flank incisions, kidneys were exteriorized and a small incision was made in the renal capsule. Spheroids/scaffold/pellets were placed under the renal capsule, and the wound was stitched up. The kidney capsule grafts were harvested 28 days after transplantation.

### Immunofluorescence analysis and image analysis

Cells were grown on glass-coverslip overnight, fixed with 4% paraformaldehyde for 15 min, washed with 1xPBS and permeabilized in 0.1% Triton X-100 for 15 min. After blocking with 1% BSA for 30 min, the samples were incubated with primary antibodies: anti-LANA (Abcam, ab4103), anti-SM22 (TAGLN, Proteintech, 10493-1-AP), anti-PDPN (Proteintech, 11629-1-AP), anti-vWF (Proteintech, 11778-1-AP), anti-CD31 (Proteintech, 11265-1-AP), anti-VCAM (Proteintech, 11444-1-AP). After washing, samples were incubated with secondary antibody Donkey anti-Rabbit IgG Alexa Fluor 555 (Life, A-31572) or Goat anti-Rat IgG Alexa Fluor 555 (Life, A-21434) for 1 hour. Images were taken using a ZEISS microscope. The same procedure was used for immunofluorescence analysis of paraffin-embedded 3D spheroids, except that 3D spheroids were first de-paraffinized, rehydrated and antigen retrieval.

Triple immunostaining for KS tissues or 3D spheroids implant sections was performed using a mouse anti-PDGFRA (Immunoway, 4G11, YM3688), anti-α-SAM (Proteintech, 67735-1-Ig) or anti-Nestin (Santa Cruz, sc-23927) antibody, a rabbit anti-PDPN (Proteintech, 11629-1-AP), anti-VEGFR2 (Proteintech, 26415-1-AP) or anti-CD31 (Proteintech, 11265-1-AP) antibody and a rat anti-LANA antibody (Abcam, ab4103). Anti-rabbit IgG Alexa 647- (Invitrogen, A-21244), Anti-mouse IgG Alexa 488- (Invitrogen, A-28175) and Anti- rat IgG Alexa 555 (Invitrogen, A-21434)-conjugated antibodies were used for secondary antibodies. Nuclei were counterstained with Hoechst 33342 (Sigma).

Digital, whole-slide fluorescence images of mIHC slides were acquired using Zeiss Axio Scan.Z1. For each KS specimen, the number of cells was counted on DAPI+ nuclei from 4–8 pictures of representative areas (180μm x 150μm) by visual scoring of color micrographs of characteristic lesions, and positive cells for every single marker and markers combination were calculated. A total of 246 to 724 cells were manually counted from every study specimen. The percentage of Nestin/ CD31, PDGFRA/PDPN or α-SAM/VEGFR2 single positive, double-positive or double-negative cells in LANA-positive were counted on 4–8 successive fields (mean: 543 cells/biopsy) per specimen manually. The number of vessels was quantified from 4–6 individual fields (354μm x 246μm) for each KS tumor sample. Fluorescence profiles of intensity signals across a line were generated to analyze the spatial association between KSHV (LANA), mesenchymal, and endothelial markers using ZEN 2.3 lite (blue edition) image analysis profile tool.

### Immunohistochemistry and Histopathology

KS tissue and 3D spheroids implant sections were deparaffinated and rehydrated. After antigen retrieval with 0.1M citrate buffer (pH 6.0), Sections were blocked with 5% BSA for 30 min. The sections were incubated with primary antibodies: anti-LANA (Abcam, ab4103), anti-human CD31 (PECAM-1, Immunoway, PT0035), anti-Ki-67 (Cell Signaling, 9129). DAB super sensitive reactivity system (Maxim Biotechnology, Fujian, KIT-9720) was used for antigen detection.

### Plasmids

The KSHV genes K1, vIL6, RTA, K8, PAN, K12, LANA, vCyclin, and vGPCR were amplified by PCR using the cDNA prepared from iSLK.219 as templates and cloned into the pMSCV-puro lentiviral vector at the Bgl II/EcoR I restriction site (Addgene plasmid # 68469). vFLIP was cloned by inserting its PCR fragment into the modified pMSCV-puro-3HA vector at the Bgl II/ Xho I restriction site. These constructs were confirmed by DNA sequencing. Lentivirus was produced by co-transfection of 293T cells with expression vector pMSCV and PIK packaging plasmid at a 1:1 ratio.

### CRISPR-Cas9-mediated knock out of KSHV vFLIP expression

Guide sequences (gRNAs) were designed to target the 5’ and 3’ regions of vFLIP using an online CRISPR design tool (http://crispr.mit.edu) and the KSHV genomic sequences (NCBI Reference Sequence: NC_009333.1). The gRNA sequences were subcloned into the BsmBI restriction site of CRISPR/Cas9 vectors lentiCRISPR v2 (Addgene plasmid # 52961). Lentivirus was produced by triple transfection of 293T cells with the sgRNA expression LentiCRISPR-v2 vector and the packaging plasmids psPAX2 (Addgene plasmid # 12260) and pMD2.G (Addgene plasmid # 12259) at a ratio of 5:3:2. PDLSCs were transduced with two gRNA/Cas9-expressing lentivirus, one for 5’ regions and the other for 3’ regions of vFLIP gene, followed by puromycin selection for 1 week.

To verify ORF71 KO efficiency, total DNA was isolated using a HiPure Tissue DNA Mini kit (Magen, D3121), and analyzed by PCR using PrimeSTAR (TaKaRa, R040A). The primers used for monitoring the efficiency of vFLIP knockout are Pr1 (ACCCTGCGTAAACAACCG) and Pr2 (ACCCAAAGACTGGCTCAT). The relative mRNA level of ORF71 was also determined by RT-qPCR using specific KSHV ORF71 primers 5′- GGATGCCCTAATGTCAATGC -3′ (forward) and 5′- GGCGATAGTGTTGGGAGTGT -3′ (reverse).

For complementation rescue experiment, synonymous mutations were introduced into the plasmid PLVX-ORF71-HA in NGG sequences of sg2 and sg3 targeted regions with primers pairs 1 and 2 (S2 Table) according to the Protocols of separate double bases site-directed mutations (Vazyme biotech co., ltd. C214). Then, the sgRNA-resistant mutant ORF71 cDNA was amplified with primers pair 3 (S2 Table) and cloned into the BamHI/ EcoRI restriction site of pLV-EF1a-IRES-Blast vector (Addgene plasmid # 85133) and verified by sequencing (S5 Fig). The sgRNA-resistant pLVEF1a-ORF71 was introduced into the PDLSCs expressing two gRNA by lentivirus transduction. After selection, the expression of vFLIP was confirmed by Western analysis.

### Statistical analysis

Statistical analyses were performed by two-tailed Student’s t-test using GraphPad prism 8.0, or the Mann-Whitney *U* test in IBM SPSS Statistics 18 software to determine the statistical significance between the experimental and control groups. The Chi^2^ tests were used to compare for differences in categorical variable distribution. *P* < 0.05 was considered statistically significant. *P<0.*05*, **P<0.*01* and ***P<0.*001*; NS, not significant (*P > 0*.*05*). Data were graphed as mean ± SEM.

## Supporting information

S1 Table Primers used for reverse-transcription real-time quantitative PCR. (PDF)

S2 Table Primers used to construct sgRNA-resistant ORF71 expression vectors. (PDF)

S1 Fig Co-expression of mesenchymal and endothelial markers in KS cells. AIDS-KS lesion tissues (lower) and their adjacent normal skin tissues (upper) were immunostained for a mesenchymal marker (PDGFRA/Nestin/α-SAM, green), an endothelial marker (PDPN/CD31/VEGFR2, red) and a KSHV marker (LANA, yellow). Nuclei were counterstained with Hoechst 33342 (blue). Scale bars, 50 μm.
(PDF)

S2 Fig Expression of viral genes RTA, K8, vIL-6, vFLIP, and vGPCR in PDLSCs. PDLSCs were transduced with the expression lentiviral vectors as indicated. RTA and K8 were detected by Western blot with specific antibodies against them. In the constructs of vIL-6, vGPCR, and vFLIP, these viral proteins were HA- or Flag-tagged, and their expression was detected with antibodies against these tags.
(PDF)

S3 Fig Confirmation of sgRNA/Cas9-mediated vFLIP-knockout in the KSHV genome. PCR products were amplified from KSHV-infected PDLSC transfected with sgRNA2 and sgRNA3 with the primers Pr1 and Pr2 (see Fig 8), TA-cloned, and sequenced. Sequence chromatography confirmed the fusion junction of gRNA 2 and gRNA 3 target sites corresponding to a deletion junction between nucleotides 122919 and 83563 from the KSHV genome, therefore verifying the deletion of vFLIP gene.
(PDF)

S4 Fig ORF71-knockout did not affect infection efficiency and the expressions of ORF71 adjacent viral genes. (A) Comparison of KSHV infectivity between control PDLSC and sgRNA-expressing PDLSC. PDLSCs and sgRNA-expressing PDLSCs were infected with GFP-KSHV in an MOI of 50 (KSHV genome equivalent) in the presence of polybrene for 48 hours and analyzed by GFP fluoresces. (B) The expression of several lytic genes (vIL6, RTA, vGPCR, ORF58, ORF59), nearby latent genes (vCyclin, LANA) and miRs (miR-K12-1, miR-K12-2) were assessed by RT-qPCR in vFLIP-KO KSHV-infected PDLSCs.
(PDF)

S5 Fig Verification of sgRNA-resistant vFLIP expression vector and its expression in vFLIP-KO KSHV-PDLSCs. (A) The sequence of sgRNA-resistant vFLIP-HA (vFLIP-sgR) that harbors synonymous mutations in NGG sequences of sg2 and sg3 targeted regions. Red # indicates the site of synonymous mutation. (B) Expression of sgRNA-resistant vFLIP-HA in vFLIP-KO KSHV-PDLSCs was verified by Western blot.
(PDF)
