# Supplementary material for: Kaposi’s sarcoma-associated herpesvirus vFLIP promotes MEndT to generate hybrid M/E state for tumorigenesis
Source: PLoS Pathog. 2021 Dec 22;17(12):e1009600. doi: 10.1371/journal.ppat.1009600 (PMC8735625; doi:10.1371/journal.ppat.1009600)
Supplement: S1 Table — (PDF) [file ppat.1009600.s001.pdf]

S1 Table Primers Used for Reverse-Transcription Real-time Quantitative PCR

**Gene**	**Forward** (5’ – 3’)	**Reverse** (5’ – 3’)
TAGLN	AGTGCAGTCCAAAATCGAGAAG	CTTGCTCAGAATCACGCCAT
α-SAM	GTGTTGCCCCTGAAGAGCAT	GCTGGGACATTGAAAGTCTCA
Nestin	CAACAGCGACGGAGGTCTC	GCCTCTACGCTCTCTTCTTTGA
PDGFRA	TGGCAGTACCCCATGTCTGAA	CCAAGACCGTCACAAAAAGGC
PDPN	AACCAGCGAAGACCGCTATAA	CGAATGCCTGTTACACTGTTGA
ICAM	ATGCCCAGACATCTGTGTCC	GGGGTCTCTATGCCCAACAA
PROX1	TGACAGCACTGATTCGGAAAATG	ATAGGCCATCAAGGGAAGGG
CD31	AACAGTGTTGACATGAAGAGCC	TGTAAAACAGCACGTCATCCTT
VEGFA	AGGGCAGAATCATCACGAAGT	AGGGTCTCGATTGGATGGCA
COL1A1	GAGGGCCAAGACGAAGACATC	CAGATCACGTCATCGCACAAC
CNN1	GAACGTGGGAGTGAAGTACGC	CAGCCCAATGATGTTCCGC
CD36	GGCTGTGACCGGAACTGTG	AGGTCTCCAACTGGCATTAGAA
VCAM	GGGAAGATGGTCGTGATCCTT	TCTGGGGTGGTCTCGATTTTA
vFLIP	GGATGCCCTAATGTCAATGC	GGCGATAGTGTTGGGAGTGT
GAPDH	GTCTCCTCTGACTTCAACAGCG	ACCACCCTGTTGCTGTAGCCAA
vIL6	TCGTTGATGGCTGGTAG	CACTGCTGGTATCTGGAA
RTA	CACAAAAATGGCGCAAGATGA	TGGTAGAGTTGGGCCTTCAGTT
vCyclin	GCTGATAATAGAGGCGGGCAATGAG	GTTGGCGTGGCGAACAGAGGCAGTC
vGPCR	AACCATCTTCTTAGATGATGAT	AATCCATTTCCAAGAACATTTA
ORF58	TGCGGAGCATTTATGGTGTA	TGCCTAAATGCCAAAAGTCC
ORF59	CGAGTCTTCGCAAAAGGTTC	AAGGGACCAACTGGTGTGAG
KSHV-miR-K12-1	CGCGCATTACAGGAAACTGGG	miR-K12-Uni-R GTGCAGGGTCCGAGGT
KSHV-miR-K12-2	CGTGCAACTGTAGTCCGGGTC	miR-K12-Uni-R GTGCAGGGTCCGAGGT
