# Supplementary material for: Kaposi’s sarcoma-associated herpesvirus vFLIP promotes MEndT to generate hybrid M/E state for tumorigenesis
Source: PLoS Pathog. 2021 Dec 22;17(12):e1009600. doi: 10.1371/journal.ppat.1009600 (PMC8735625; doi:10.1371/journal.ppat.1009600)
Supplement: S2 Table — (PDF) [file ppat.1009600.s002.pdf]

S2 Table Primers Used to Construct sgRNA-resistant ORF71 Expression Vectors

**Primer**	**Forward** (5' – 3')	**Reverse** (5' – 3')
Pair 1	GATGACAG**A**GAAGTGGTATTGTTCCTCCTAAACGTGT	ATAGTGTTG**A**GAGTGTGATGGGCCGGAAAGG
Pair 2	TCACACTC**T**CAACACTATCGCCATACACCATACCC	TACCACTTC**T**CTGTCATCCGTGCCCAGTTTC
Pair 3	TTCAGGTGTCGTGAGGATCCATGGCCACTTACGAGGTTCTCT	CGGCCGCCCTCGAGGAATTCCTAAGCGTAGTCTGGGACGTCG
