# Supplementary figures and images for: Kaposi’s sarcoma-associated herpesvirus vFLIP promotes MEndT to generate hybrid M/E state for tumorigenesis
Source: PLoS Pathog. 2021 Dec 22;17(12):e1009600. doi: 10.1371/journal.ppat.1009600 (PMC8735625; doi:10.1371/journal.ppat.1009600)

A

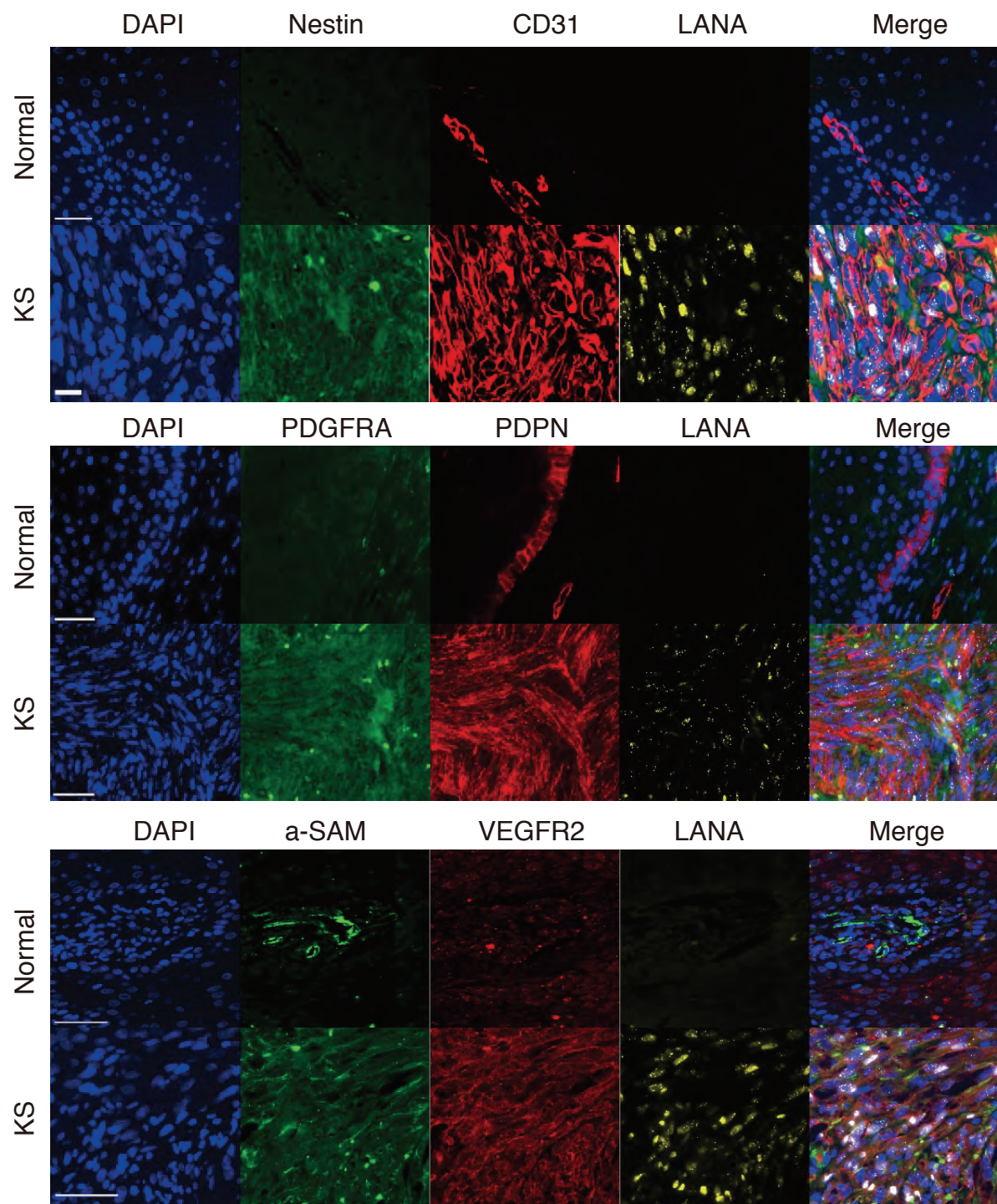

B

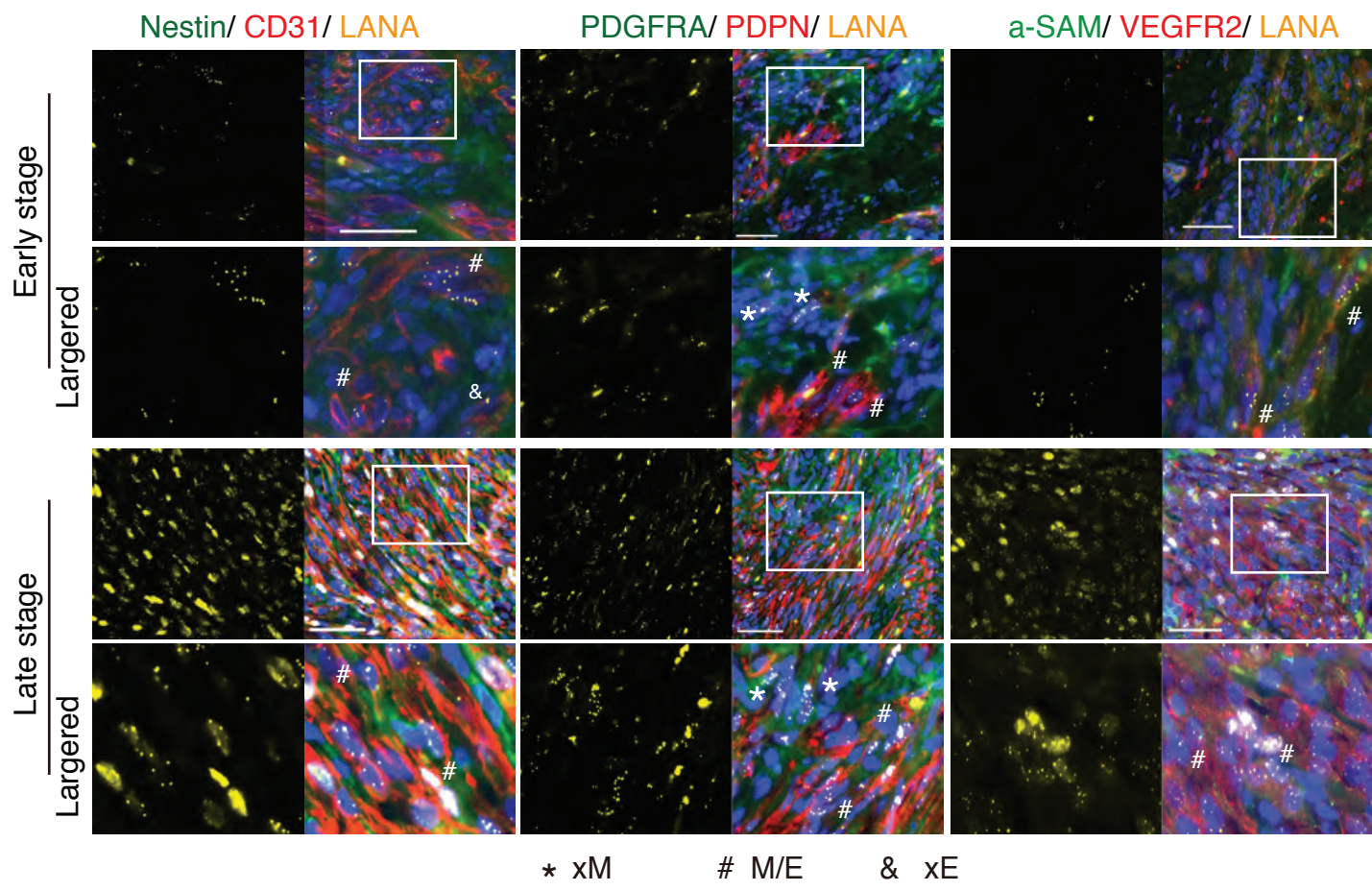

Supplement: S1 Fig — AIDS-KS lesion tissues (lower) and their adjacent normal skin tissues (upper) were immunostained for a mesenchymal marker (PDGFRA/Nestin/α-SAM, green), an endothelial marker (PDPN/CD31/VEGFR2, red) and a KSHV marker (LANA, yellow). Nuclei were counterstained with Hoechst 33342 (blue). Scale bars, 50 μm. (PDF) [file ppat.1009600.s003.pdf]

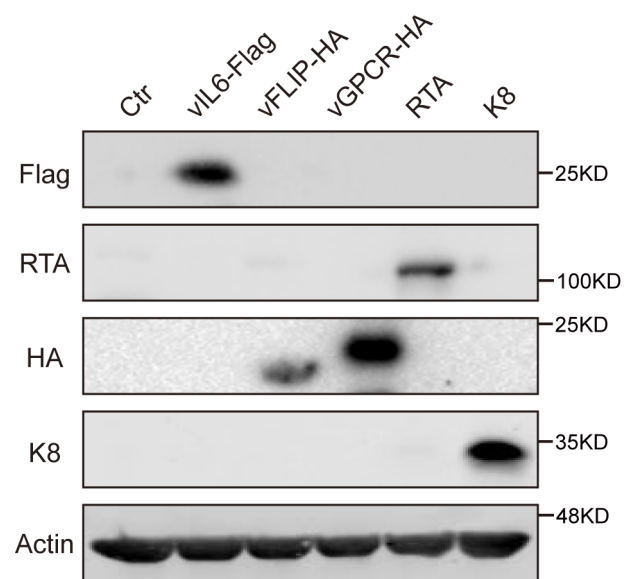

Supplement: S2 Fig — PDLSCs were transduced with the expression lentiviral vectors as indicated. RTA and K8 were detected by Western blot with specific antibodies against them. In the constructs of vIL-6, vGPCR, and vFLIP, these viral proteins were HA- or Flag-tagged, and their expression was detected with antibodies against these tags. (PDF) [file ppat.1009600.s004.pdf]

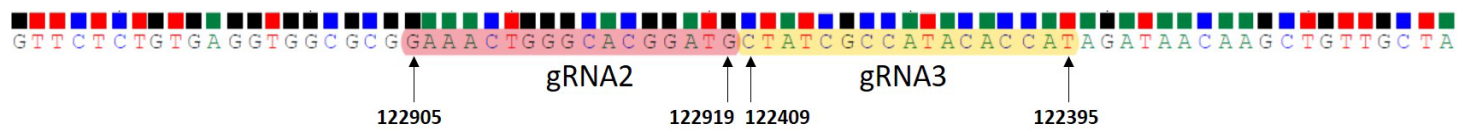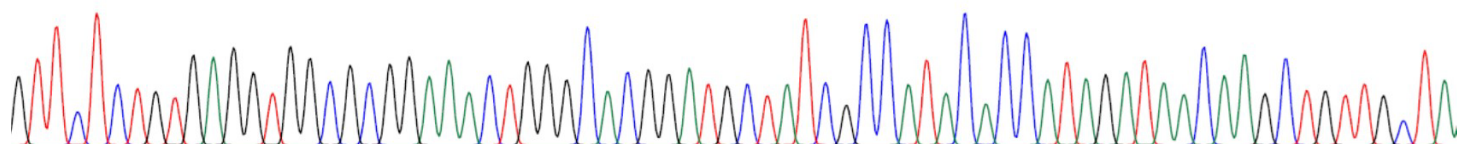

Supplement: S3 Fig — PCR products were amplified from KSHV-infected PDLSC transfected with sgRNA2 and sgRNA3 with the primers Pr1 and Pr2 (see Fig 8), TA-cloned, and sequenced. Sequence chromatography confirmed the fusion junction of gRNA 2 and gRNA 3 target sites corresponding to a deletion junction between nucleotides 122919 and 83563 from the KSHV genome, therefore verifying the deletion of vFLIP gene. (PDF) [file ppat.1009600.s005.pdf]

A

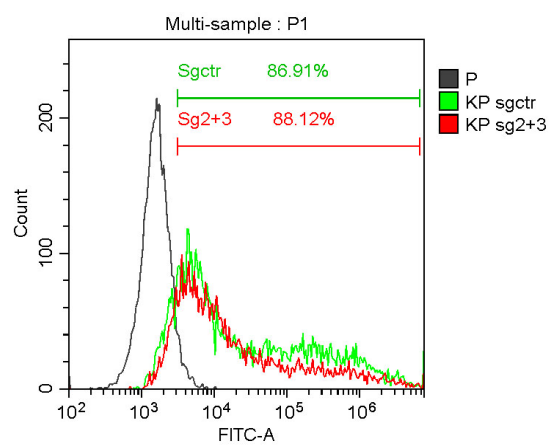

B

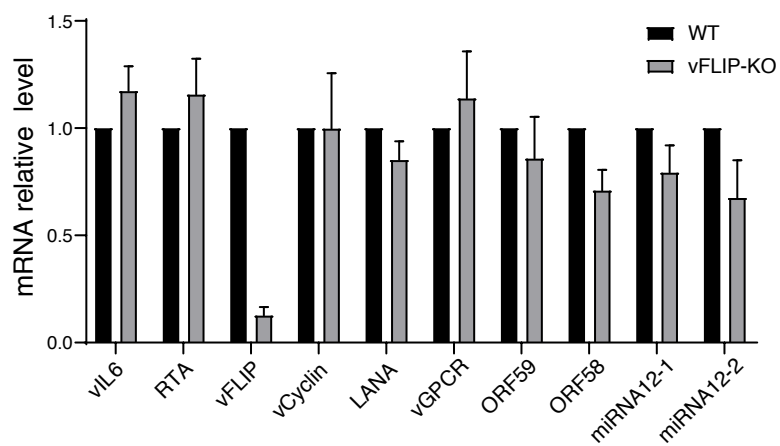

Supplement: S4 Fig — (A) Comparison of KSHV infectivity between control PDLSC and sgRNA-expressing PDLSC. PDLSCs and sgRNA-expressing PDLSCs were infected with GFP-KSHV in an MOI of 50 (KSHV genome equivalent) in the presence of polybrene for 48 hours and analyzed by GFP fluoresces. (B) The expression of several lytic genes (vIL6, RTA, vGPCR, ORF58, ORF59), nearby latent genes (vCyclin, LANA) and miRs (miR-K12-1, miR-K12-2) were assessed by RT-qPCR in vFLIP-KO KSHV-infected PDLSCs. (PDF) [file ppat.1009600.s006.pdf]

A

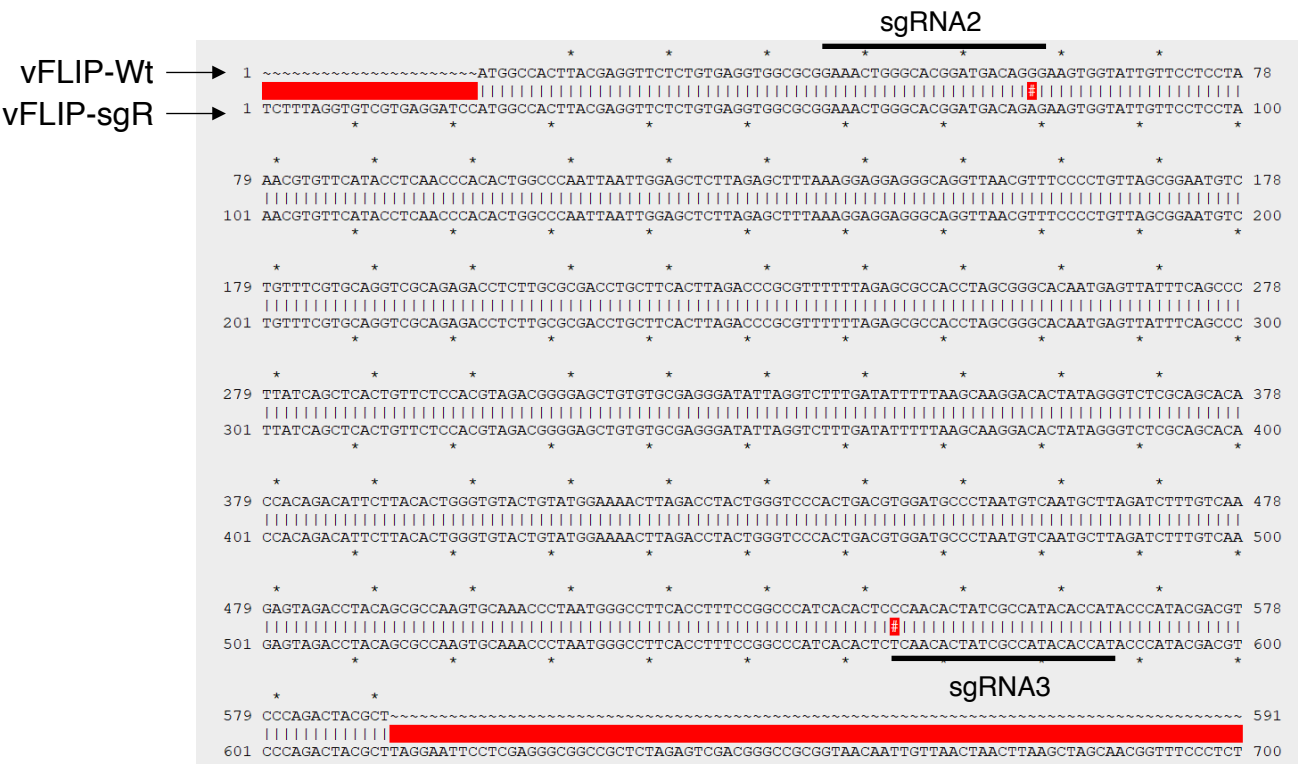

B

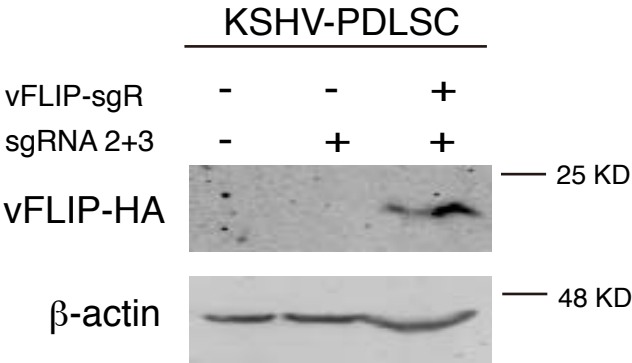

Supplement: S5 Fig — (A) The sequence of sgRNA-resistant vFLIP-HA (vFLIP-sgR) that harbors synonymous mutations in NGG sequences of sg2 and sg3 targeted regions. Red # indicates the site of synonymous mutation. (B) Expression of sgRNA-resistant vFLIP-HA in vFLIP-KO KSHV-PDLSCs was verified by Western blot. (PDF) [file ppat.1009600.s007.pdf]
